# Retinoic acid alleviates manganese-induced neurotoxicity in SH-SY5Y cells via the Trim28/α-synuclein/H4K91ac epigenetic axis

**DOI:** 10.3389/fphar.2026.1883748

**Published:** 2026-08-25

**Authors:** Chunyan Ao, Hao Liu, Ruyun Ban, Tao Huang, Yanxu Xiao, Wei Chen, Yao Feng, Hua Zhao, Jiaqi Ban, Jun Li

**Affiliations:** School of Public Health, Key Laboratory of Environmental Pollution Monitoring and Disease Control, Ministry of Education, Guizhou Medical University, Guiyang, China

**Keywords:** dopaminergic dysfunction, histone acetylation, manganese neurotoxicity, retinoic acid, Trim28/α-synuclein axis

## Abstract

**Background:**

Manganese (Mn)-induced neurotoxicity is a major public health concern. The aim of this study is to assess whether retinoic acid, a metabolite of vitamin A, can counteract manganese-induced neurotoxicity in SH-SY5Y cells by counteracting epigenetic dysregulation.

**Methods:**

*In vivo*, we assessed neurobehavioral function, striatal pathology, synaptic ultrastructure, Mn content, dopamine levels, and expression of Trim28, α-synuclein (α-syn), H4K91ac, and DAT in Mn exposed rats. *In vitro*, we investigated the interactions between α-syn, Trim28 and H4 using immunofluorescence, molecular docking and immunoprecipitation techniques. We used chromatin immunoprecipitation to detect the enrichment of H4K91ac within the DAT promoter region. Additionally, we knocked down α-syn and Trim28 using siRNA to explore their potential molecular mechanisms.

**Results:**

Mn exposure induced behavioral deficits, synaptic damage, and dopaminergic dysfunction in rats. Mechanistically, Trim28 mediated α-syn nuclear translocation. Nuclear α-syn bound histone H4, reducing H4K91ac enrichment at the DAT promoter, suppressing DAT expression, and disrupting dopamine homeostasis. Knockdown of Trim28 or α-syn reversed these effects. Retinoic acid inhibits the nuclear translocation of α-syn by modulating Trim28, and restores the levels of H4K91ac and DAT, thereby alleviating manganese-induced neurotoxicity in cells.

**Conclusion:**

This study elucidates the role of the Trim28/α-syn/H4K91ac axis in manganese-induced dopaminergic dysfunction and identifies Trim28 as a potential target for retinoic acid.

## 1 Introduction

Global environmental pollution is becoming increasingly severe, with one of the primary causes being exposure to heavy metals (Zhang et al., 2022). Manganese (Mn), the fifth most abundant metal on Earth, finds extensive application across various sectors, yet it concurrently functions as an environmental pollutant (Kwakye et al., 2015). In addition to occupational exposure, manganese exposure is also prevalent in environmental media such as air and water (Wang et al., 2010; Markiv et al., 2023). The impact of environmental manganese pollution on human health has become a major public health issue. Long-term exposure to high concentrations of manganese in the environment can induce neurotoxicity (Wu et al., 2022), but the underlying molecular mechanisms remain unclear. Disrupted dopamine (DA) homeostasis is a key factor in manganese neurotoxicity. DA, the brain’s most abundant catecholamine neurotransmitter, transmits signals via postsynaptic DA receptors after release into the synaptic cleft. The DAT on the presynaptic membrane then reabsorbs most DA for reuse (Lebowitz and Khoshbouei, 2020), making DAT a crucial regulator of DA levels. DAT dysfunction is linked to neurological disorders like Parkinson’s disease (DiCarlo et al., 2019). Research indicates that manganese exposure can reduce DAT expression, leading to dysfunction, but the mechanism behind this downregulation is not well understood (Saputra et al., 2016; Conley et al., 2020).

Histone acetylation is one of the epigenetic modification methods that regulate gene transcription and expression. Its homeostasis imbalance is closely related to the neurodegenerative process of neurodegenerative diseases (Toker et al., 2021). Our team previously conducted a quantitative acetylated proteomics study on the striatum of rats with subchronic manganese exposure using LC-MS/MS technology, and found that the acetylation level of lysine at position 91 of histone H4 (H4K91ac) was significantly reduced (Ao et al., 2024). Moreover, we have confirmed that manganese exposure can reduce the level of H3K27ac in the promoter region of oxidative stress-related genes, inhibit their expression, and cause oxidative damage in rats and cells (Yang et al., 2022; Liu et al., 2024b). Therefore, the downregulation of histone acetylation levels, which inhibits gene transcription and expression, may be a key factor in manganese-induced neurotoxicity. Does H4K91ac regulate DAT expression and participate in the pathogenesis of manganese?

While histone acetylation dysregulation is implicated in neurodegenerative diseases like manganese poisoning, the underlying cause remains unclear. α-syn accumulation in the brain is neurotoxic, particularly when it occurs in the nucleus (Zhou et al., 2013; Ma et al., 2023; Pan et al., 2023). Nuclear α-syn accumulation induces DNA damage, apoptosis, and inflammation in mouse hippocampal neurons, leading to motor and cognitive dysfunction (Pan et al., 2022). α-Syn overexpression also alters genome-wide DNA methylation and hydroxymethylation, causing transcriptional dysregulation, suggesting a link between α-syn nuclear translocation and epigenetic changes (Schaffner et al., 2022). Tripartite motif-containing protein 28 (Trim28), a transcriptional cofactor primarily located in the nucleus, can promote the nuclear translocation of proteins (Wang et al., 2013; Wang et al., 2019; Czerwińska et al., 2017). Although Trim28 has been extensively studied in cancer, its role in neurodegenerative diseases is not well-understood. Rousseaux et al. have demonstrated that Trim28 is a key nuclear transport chaperone for α-synuclein (Rousseaux et al., 2016). However, the role of this molecular axis in manganese-induced neurotoxic damage, and whether it mediates neurotoxicity by influencing epigenetics, has not yet been investigated.

Effective treatments for manganese poisoning are lacking. Retinoic acid, a vitamin A derivative with anti-inflammatory, anti-apoptotic, and neuroprotective properties (Kang and Koh, 2022), shows promise as a novel therapeutic for neurodegenerative diseases (Clark et al., 2020). As a transcriptional regulator, retinoic acid uniquely induces neurite outgrowth, promotes neuronal survival, and regulates protein homeostasis. Studies indicate that retinoic acid signaling can delay Alzheimer’s disease progression by modulating AD-related gene expression, inhibiting amyloid precursor protein processing, and reducing Tau phosphorylation (Zhang et al., 2023). Moreover, retinoic acid-loaded polymer nanoparticles have shown protective effects on dopaminergic neurons in a Parkinson’s disease model (Esteves et al., 2015). These findings support retinoic acid’s potential in treating neurodegenerative diseases.

This study investigates how manganese neurotoxicity disrupts histone acetylation and DA homeostasis. We found that α-syn nuclear translocation, mediated by Trim28, induces histone acetylation dysregulation and DA imbalance, leading to neurological damage. Furthermore, we explored the neuroprotective potential of retinoic acid.

## 2 Materials and methods

### 2.1 Chemicals and antibodies

MnCl_2_·4H_2_O (purity ≥99%, M813684) was purchased from Shanghai Macklin Biochemical Co., Ltd., retinoic acid (purity ≥98%, catalogue number R2625) was purchased from Sigma-Aldrich, anti-H4 (16047-1-AP), anti-β-actin (66009-1-Ig), anti-DAT (22524-1-AP), anti-Trim28 (15202-1-AP), anti-Lamin B1 (12987-1-AP),and anti-β-tubulin (10094-1-AP) were all from Wuhan Sanying Biotechnology Co., Ltd., H4K91ac (ab4627) antibody was purchased from Abcam, α-synuclein (sc53955) antibody was from Santa Cruz, the chromatin immunoprecipitation kit was provided by Cell Signaling Technology, the immunoprecipitation kit was purchased from Shanghai Biyuntian Biotechnology Co., Ltd., and the DA enzyme-linked immunosorbent assay kit was from Yilai Rui Te Biotechnology Co., Ltd.

### 2.2 Animals and grouping

Twenty-four male Sprague-Dawley rats (180–220 g) were purchased from the Laboratory Animal Centre of Guizhou Medical University. The rats were acclimatised and fed for 1 week in an animal housing facility maintained at a temperature of 18 °C–22 °C and a humidity of 40–70 per cent, with a 12-h light-dark cycle. Using a random number table, rats were assigned to four groups (n = 6 per group) based on body weight: control (sterile distilled water), low-Mn (6 mg/kg MnCl_2_·4H_2_O), medium-Mn (12 mg/kg), and high-Mn (18 mg/kg), administered intraperitoneally once daily, 5 days per week, for 18 weeks. The selected doses were derived by extrapolation from the occupational exposure limit for manganese and its compounds (0.15 mg/m^3^ as the permissible concentration-time weighted average specified in GBZ 2.1–2019), with conversion based on standard adult 8-h breathing volume and reference body weight. Male rats were used because occupational manganese exposure primarily affects males, and previous animal studies have predominantly used males to ensure comparability. Predefined exclusion criteria included: non-experimental infections, >20% weight loss, peritonitis, or unexpected death. Humane endpoints were: >20% weight loss, inability to feed/drink, or moribund state.

At the end of the 18-week exposure period, rats were deeply anesthetized with intraperitoneal 2% sodium pentobarbital; anesthetic depth was confirmed by loss of toe-pinch and corneal reflexes. Blood was collected by cardiac puncture via thoracotomy, followed by rapid decapitation. Brains were immediately removed, striata dissected on dry ice, frozen in liquid nitrogen, and stored at −80 °C. Death was confirmed by exsanguination and cardiac arrest. All procedures complied with the ARRIVE guidelines (Percie du Sert et al., 2020) and were approved by the Animal Care and Use Committee of Guizhou Medical University (Approval Nos. 1900218, 2400043).

### 2.3 Behavioral tests

All behavioural tests were conducted in a blinded manner and were carried out and analysed by experimenters who were unaware of the group allocations.

#### 2.3.1 Balance beam

The test method refers to our previous study (Yang et al., 2022). Briefly, the score is based on the number of times the rat’s forelimbs and hindlimbs slip off the balance beam. Each rat is tested 3 times, and the average score is taken.

#### 2.3.2 Eight-arm maze

Food was placed at the end of each arm and in the central area of the maze, allowing the rats to feed freely for 10 min. After 2 days of training, 4 arms were randomly selected as food arms and 4 arms as empty food arms, allowing the rats to explore freely until they finished eating the food or the total exploration time reached 10 min, repeating the training for 5 days. On the 8th day, one food pellet was placed in each arm, and the animals were placed in the center of the maze, allowing them to move freely in the maze and ingest the food pellets until the animals had eaten all the food pellets in the designated 4 food arms. If the food pellets were not finished after 10 min, the experiment was terminated, and their free exploration trajectory was recorded.

### 2.4 Hematoxylin-eosin staining

The striatum was fixed in 4% paraformaldehyde, dehydrated in graded ethanol solutions, and embedded before sectioning. Sections were stained with hematoxylin and eosin, sealed with neutral balsam, and imaged using a digital slide scanner.

### 2.5 Transmission electron microscopy

The striatum was prefixed in 3% glutaraldehyde and post-fixed in 1% osmium tetroxide. After dehydration with acetone, the samples were embedded. Sections were stained with uranyl acetate and lead citrate and observed under a JEM-1400FLASH transmission electron microscope.

### 2.6 Inductively coupled plasma mass spectrometry

The manganese content in the rat striatum was determined using inductively coupled plasma mass spectrometry (ICP-MS; Agilent 7850). Striatal tissues (10 mg) were placed in a high-pressure digestion vessel with 4 mL of 65% HNO_3_ and 1 mL of 30% H_2_O_2_ and digested overnight. Subsequently, samples were dried at 160 °C for 4 h. The residue was transferred to a volumetric flask, diluted to 10 mL with 1% nitric acid, and analysed. Scandium (Sc), yttrium (Y), and indium (In) were used as internal standards. The concentrations of the standard solutions were 0, 0.25, 0.5, 2, 10, 20, 40 and 100 μg/L.

### 2.7 Liquid chromatography-mass spectrometry (LC-MS)

The DA content in the striatum was determined using a Triple Quad 5500+. After weighing the striatum, a volume of 0.2% formic acid in methanol (1:1) equal to 10 times the volume of the sample was added, and the mixture was ultrasonically homogenised in an ice bath. Take 200 μL of the homogenate, add 0.4 mL of ice-cold 0.2% formic acid in methanol, mix thoroughly, and leave to stand at 4 °C for 30 min. Centrifuge at 4 °C (12,000 rpm, 5 min), transfer the supernatant to a sample vial after filtration, and analyse on the instrument.

Chromatographic conditions: Ultimate XB-C18 column (100 mm × 2.1 mm, 2.6 μm), column temperature 40 °C. Mobile phase A was water containing 0.1% formic acid; mobile phase B was acetonitrile containing 0.1% formic acid; flow rate 0.2 mL/min. The elution programme was as follows: 0–0.5 min, 5% B; 0.5–4 min, 40% B; 4–5 min, 95% B; 5–6 min, 95% B; 6–6.1 min, 5% B; 6.1–10 min, 5% B. The m/z value of DA was 154→137. Ion source parameters: spray voltage for positive ions 5500 V, for negative ions −4500 V; ion source temperature 500 °C; gas 1 45 psi, gas 2 50 psi; curtain gas (CUR) 20 psi; dwell time 50 ms. Multiple Reaction Monitoring (MRM) transition and compound parameters: forward bias (EP) 15 V, reverse bias (CXP) 6 V, declustering potential (DP) 69 V, collision energy (CE) 16 eV.

### 2.8 Cell culture

Human neuroblastoma cells (SH-SY5Y cells) were purchased from the Cell Bank of Wuhan University. SH-SY5Y cells were authenticated by STR profiling and used within passage 5–20. Cells were cultured in DMEM supplemented with 10% fetal bovine serum (Gibco, USA) and 1% antibiotics and incubated at 37 °C in a 5% CO_2_ incubator. MnCl_2_·4H_2_O was prepared in sterile pure water at concentrations of 0, 100, 200, and 400 μmol/L. Prepare a retinoic acid stock solution (20 mM) using dimethyl sulphoxide (final concentration 0.1%), and aliquot it for storage at −80 °C. When the cells were in the logarithmic growth phase, they were treated with different concentrations of MnCl_2_·4H_2_O. For the retinoic acid treatment group, the cells were first pre-treated with retinoic acid (20 μM) for 72 h. The culture medium was then replaced with complete medium containing either manganese or no manganese, and the cells were harvested after a further 24 h of culture.

### 2.9 CCK8 assay for cell viability

SH-SY5Y cells were seeded in 96-well plates at a density of 1 × 10^4^ cells/well. After the cells adhered and reached approximately 80% confluence, 100 μL of the test substance was added to each well. After incubation for a period of time, 10 μL of CCK-8 solution was added to each well and incubated for 4 h. The absorbance values were measured at a wavelength of 450 nm using a microplate reader.

### 2.10 SiRNA transfection

2.5 × 10^6^ cells were seeded in 6-well plates with 2 mL of complete antibiotic-free medium to achieve a cell density of approximately 30%–50% at the time of transfection. 5 μL of 20 μM siRNA duplex was diluted in 200 μL of serum-free medium and mixed thoroughly. Subsequently, 5 μL of RNAFit was added, followed by incubation at room temperature for 10 min. After medium exchange, the resulting 200 μL transfection complex was added to a 6-well plate and mixed thoroughly. The final volume of medium per well was 2 mL, resulting in a final siRNA concentration of 50 nM. The plate was incubated in a 37 °C, 5% CO_2_ incubator. Transfection efficiency of siRNA was detected by Western Blot.

### 2.11 Quantitative reverse transcription PCR (RT-qPCR)

Total RNA was extracted using the Trizol method, and RNA concentration and OD values were measured on a microplate reader. Reverse transcription was carried out using a reverse transcription kit under the following conditions: 42 °C for 30 min, 75 °C for 15 min, and 4 °C for 2 min. Using the cDNA as a template, quantitative polymerase chain reaction (qPCR) was performed on a Bio-Rad CFX96 system. The reaction mixture consisted of 5 μL TB Green® Premix Ex Taq™ II, 0.5 μL each of forward and reverse primers, 1 μL cDNA template, and 3 μL sterile water. The thermal cycling programme was as follows: 3 min pre-denaturation at 95 °C, denaturation at 95 °C for 10 s, annealing at 55 °C for 30 s, and extension at 60 °C for 20 s, for a total of 40 cycles; following amplification, a melting curve analysis was performed (65 °C–95 °C, with a temperature increase of 0.5 °C per step, each step lasting 5 s). GAPDH was used as an internal control to calculate the relative mRNA expression levels of each gene. GAPDH was used as an internal reference to calculate the relative mRNA expression levels of the genes. The primer sequences for each gene are listed in Supplementary Table S1.

### 2.12 Extraction of nuclear proteins and cytoplasmic proteins

Cytoplasmic proteins: Scrape the cells, add 100 μL of cytoplasmic protein extraction reagent A containing protease inhibitors, and vortex to ensure the cell pellet is completely dispersed. After incubating on ice for 10 min, add 5 μL of cytoplasmic protein extraction reagent B, vortex for 5 s, incubate on ice for 1 min, then vortex for a further 5 s. Next, centrifuge at 16,000 g at 4 °C for 5 min, and transfer the supernatant to a new centrifuge tube. Nuclear proteins: Add 50 μL of nuclear protein extraction reagent containing protease inhibitors to the pellet, vortex for 15 s, incubate on ice for 2 min, then vortex for a further 15 s, repeating this cycle for a total of 30 min. Finally, centrifuge at 16,000 g at 4 °C for 10 min, and transfer the supernatant to a new centrifuge tube.

### 2.13 Western blot (WB)

Proteins were extracted and quantified using bicinchoninic acid (BCA). 20 μg of protein was separated using sodium dodecyl sulfate-polyacrylamide gel electrophoresis and transferred to polyvinylidene difluoride membranes. Subsequently, the membranes were blocked with 5% skim milk at room temperature for 2 h and incubated with the following primary antibodies at 4 °C overnight: H4 (1:600), H4K91ac (1:1000), Trim28 (1:2000), α-synuclein (1:500), DAT (1:1000), β-actin (1:20000), Lamin B1 (1:10000), β-tubulin (1:6000). After washing, the membranes were incubated with HRP-conjugated secondary antibodies for 2 h. Images were acquired using a gel imaging system (Bio-Rad, USA), and band gray values were analyzed using ImageJ software. Normalization was performed using H4, β-actin, Lamin B1, and β-tubulin as internal references.

### 2.14 Immunofluorescence

Tissue sections were dewaxed with xylene and hydrated in a graded series of ethanol. After antigen retrieval for 20 min, the sections were blocked with 10% goat serum. For cells, cell slides were first fixed with 4% paraformaldehyde and then permeabilized with 0.1% Triton X-100. After removing Triton X-100, goat serum was added for blocking. Samples were incubated with the following primary antibodies: Trim28, α-synuclein, H4, at 4 °C overnight. Then, the samples were incubated with secondary antibodies at room temperature for 2 h, and finally, mounted with a mounting medium containing DAPI. Images were observed and acquired using a confocal microscope.

### 2.15 Co-immunoprecipitation

Normal mouse IgG antibody and target antibodies were added to the extracted cytoplasmic and nuclear proteins, respectively, and incubated on a rotator at 4 °C overnight. Protein samples were incubated with protein A/G Plus-Agarose on a rotator at 4 °C overnight. After centrifugation, the agarose was washed with 1xIP and 0.1xIP buffer, and after another centrifugation, 1x loading buffer was added and heated in a 95 °C metal bath for 5 min. After centrifugation, the supernatant was collected as the immunoprecipitate.

### 2.16 Chromatin immunoprecipitation

The assay was performed according to a previous method (Liu et al., 2024a). Briefly, after cross-linking with 37% formaldehyde, the samples were digested with micrococcal nuclease and sonicated. IgG and H4K91ac antibodies were added to the samples separately and incubated at 4 °C overnight. Protein G magnetic beads were added to the samples and incubated for 2 h. After washing with low-salt and high-salt buffers, the chromatin was eluted and de-crosslinked, and finally, DNA was purified using a spin column for qPCR. Calculation of CHIP-qPCR enrichment efficiency: Enrichment percentage = 2% × 2(^C[T]^_Input Sample-_^C[T]^_IP Sample_). Two pairs of primers were designed for the promoter region of the DAT gene, and the primer sequences are listed in Supplementary Table S2.

### 2.17 Enzyme-linked immunosorbent assay

After the end of the treatment, cell culture supernatants were collected. Standard and test samples were added to the standard wells and sample wells, respectively. Biotinylated antibody working solution was added, and the mixture was incubated at 37 °C for 45 min. After washing, enzyme working solution was added, and the mixture was incubated at 37 °C for 30 min. After washing again, substrate solution was added and incubated in the dark for 20 min. Finally, stop solution was added, and the absorbance was measured at a wavelength of 450 nm.

### 2.18 Cellular thermal shift assay (CETSA)

Cells were collected by centrifugation at 4 °C and 1,500 rpm for 5 min. They were then resuspended in PBS containing protease inhibitors, divided equally among tubes, and incubated with RA or DMSO at 37 °C for 3 h. Samples from each group were heated for 3 min at different temperatures (37 °C–62 °C). Following five freeze-thaw cycles, the supernatant was collected by centrifugation and then detected using Western blot. Non-linear regression (Boltzmann sigmoidal fitting) was used to derive the melting temperatures (Tm) for each group and to compare them.

### 2.19 Network pharmacology analysis

The structure and Canonical SMILES of retinoic acid were obtained from PubChem (https://pubchem.ncbi.nlm.nih.gov/). Retinoic acid-related targets were obtained from the CTD database (https://ctdbase.org/) and the SwissTargetPrediction database (http://swisstargetprediction.ch/), duplicates were removed following the merging of these datasets. Potential targets for neurodegenerative diseases were sourced from the GeneCards database (https://www.genecards.org/) and the OMIM database (https://www.omim.org). For the GeneCards targets, a threshold of a relevance score ≥ the median of 4.63 was applied; duplicates were removed after the two datasets were merged. Subsequently, a Venn diagram was drawn using the microbioinformatics platform (https://www.bioinformatics.com.cn/), and 3404 overlapping targets were identified.

The intersection targets were imported into the NetworkAnalyst (https://www.networkanalyst.ca/) platform, with “*Homo sapiens*” selected as the species, to obtain the initial PPI network graph. The confidence threshold was set to 0.9, and the top 500 genes were filtered based on their Degree values. The final PPI network graph was visualized using Cytoscape version 3.10.0 software.

GO functional enrichment analysis and KEGG pathway enrichment analysis were performed on 500 key targets using the DAVID database (https://davidbioinformatics.nih.gov/). The entire human genome was used as the background gene set, and the Benjamini–Hochberg method was employed for multiple comparison correction, with an FDR <0.05 set as the significance threshold. The analysis results were visualised using bubble plots generated via the Microbioinformatics platform.

### 2.20 Molecular docking

The relevant protein codes were obtained from the UniProt database (https://www.uniprot.org/), and the three-dimensional structures of the proteins were obtained from the AlphaFold database (https://www.alphafold.ebi.ac.uk/) using these codes. The resulting data was placed in the Gramm (https://gramm.compbio.ku.edu/request) platform for docking prediction. The detailed information of the protein molecule docking interaction surface was displayed using the PDBePISA database (https://www.ebi.ac.uk/pdbe/pisa/), and finally, Pymol software was used to visualize the protein-protein docking.

For the molecular docking between retinoic acid and Trim28, AMDock 1.5.2 software was used. The 2D structure of retinoic acid was downloaded from Pubchem, converted to a 3D structure using Open Babel software, and the PDB format was downloaded. Subsequently, the PBD format of Trim28 and retinoic acid was placed in AMDock software for molecular docking. All docking parameters were kept at default value. The Vina scoring function was used to calculate binding affinities (kcal/mol). And then Pymol software was used to visualize the small molecule-protein docking.

### 2.21 Statistical analysis

Data analysis was performed using SPSS 26.0 and GraphPad Prism 9.0. Data were expressed as mean ± standard deviation (SD). For multi-group comparisons conforming to a normal distribution with homogeneity of variances, one-way ANOVA followed by Tukey’s *post hoc* test was used. It should be noted that these analyses test predefined group differences and do not formally establish interaction effects (e.g., Mn × siRNA or Mn × retinoic acid interaction), as the experimental design was not powered for factorial analysis. For comparisons between two groups with a normal distribution, t-tests were performed; Pearson’s correlation analysis was used for correlation analysis. For *in vivo* studies, n represents independent animals; for *in vitro* studies, n represents independent cell culture experiments. *P* < 0.05 was considered statistically significant.

## 3 Results

### 3.1 Manganese exposure causes striatal synaptic damage and neurobehavioral abnormalities in rats

**FIGURE 1:**
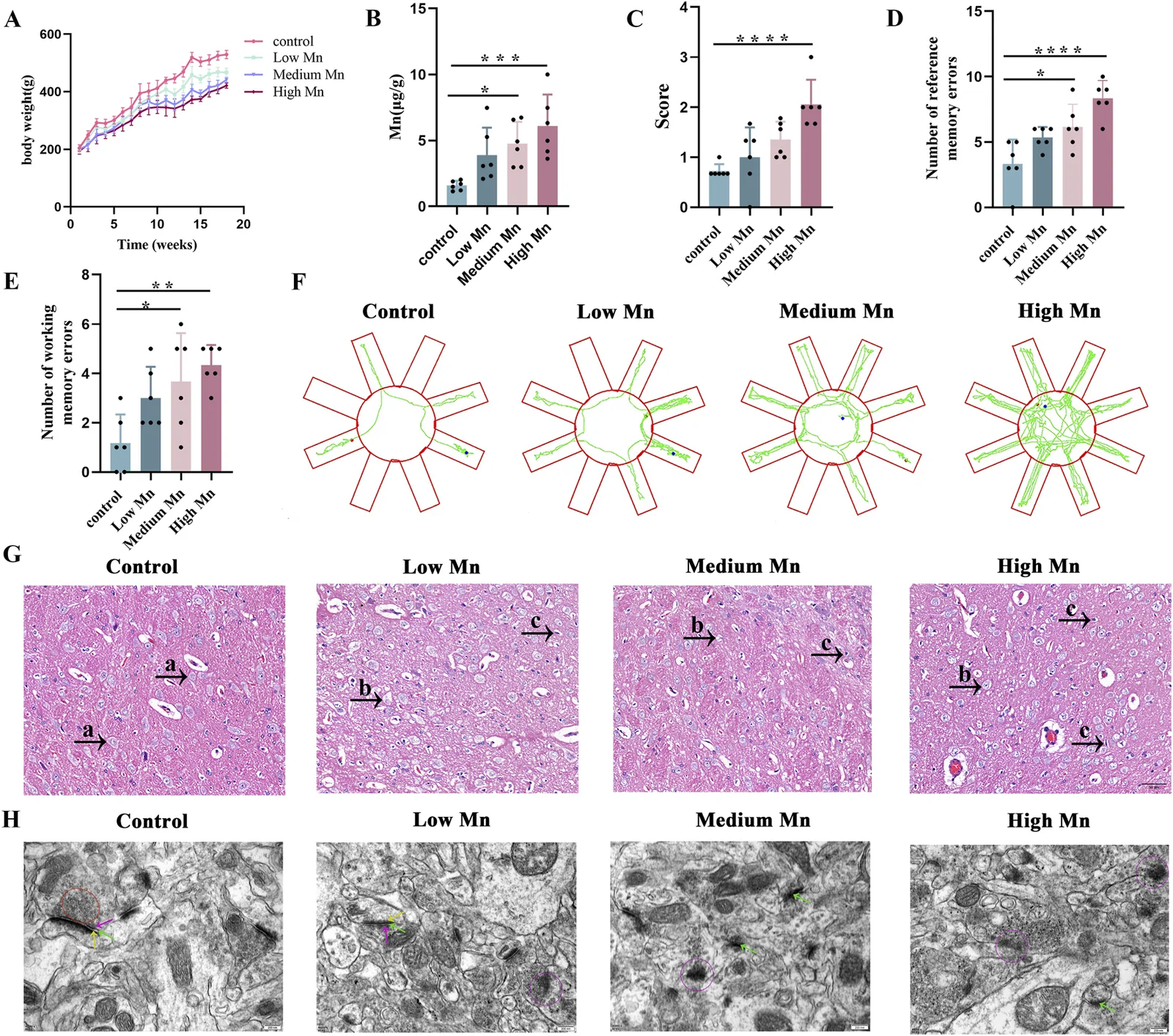
Striatal Synaptic Injury and Neurobehavioral Abnormalities in Rats Induced by Manganese. **(A)** Changes in body weight of rats exposed to different doses of manganese (n = 6). **(B)** ICP-MS determination of manganese levels in the striatum (n = 6). One-way ANOVA: F (3, 20) = 6.702, *p* = 0.0026. Post hoc Tukey’s test: Control vs. Medium Mn, mean difference 95% CI [−5.807, −0.540]; Control vs. High Mn, mean difference 95% CI [−1.883, −1.750]. **(C)** Balance beam test scores (n = 6). One-way ANOVA: F (3, 20) = 10.76, *p* = 0.0002. Post hoc Tukey’s test: Control vs. High Mn, mean difference 95% CI [−1.996, −0.701]. **(D,E)** Reference memory and working memory error counts (n = 6). One-way ANOVA: reference memory, F (3, 20) = 11.47, *p* = 0.0001; working memory, F (3, 20) = 5.948, *p* = 0.0045. Post hoc Tukey’s test: Control vs. Medium Mn, mean difference 95% CI [−5.029, −0.637] (reference) and [−4.508, −0.492] (working); Control vs. High Mn, mean difference 95% CI [−7.196, −2.804] (reference) and [−5.175, −1.158] (working). **(F)** Behavioral trajectory map of rats in the eight-arm maze. **(G)** HE staining of striatal neurons (×400), scale bar: 50 μm. a: normal neuron; b: vacuolar degeneration; c: neuronal necrosis. **(H)** Transmission electron microscopy of striatal synaptic structure (×15,000), scale bar: 200 nm. Purple arrows: presynaptic membrane; green arrows: synaptic cleft; yellow arrows: postsynaptic membrane; red circles: synaptic vesicles; purple circles: blurred synaptic structure. Data are expressed as mean ± SD. *p < 0.05, **p < 0.01, ***p < 0.001, ****p < 0.0001 vs. control group.

In rats, increasing manganese exposure correlated with reduced weight gain (Figure 1A). Given manganese’s neurotoxicity, we measured striatal manganese content and confirmed its accumulation in this tissue (Figure 1B). Neurobehavioral tests revealed that manganese exposure increased balance beam scores (Figure 1C) and reference/working memory errors (Figures 1D–F), suggesting impaired learning, memory, and motor function due to striatal manganese accumulation. HE staining revealed morphological features suggestive of manganese-induced vacuolar degeneration and necrosis in the striatum (Figure 1G). Furthermore, transmission electron microscopy further showed that manganese-exposed rats exhibited marked disruption of synaptic structural integrity, characterized by blurring or loss of the normal presynaptic and postsynaptic membrane profiles and a disordered, uneven appearance of synaptic vesicles, whereas control groups exhibited normal synaptic morphology (Figure 1H). These findings demonstrate that manganese damages synaptic structure in rats, leading to learning, memory, and motor dysfunction.

### 3.2 Manganese disrupts DA balance by reducing H4K91ac levels in the DAT promoter region

**FIGURE 2:**
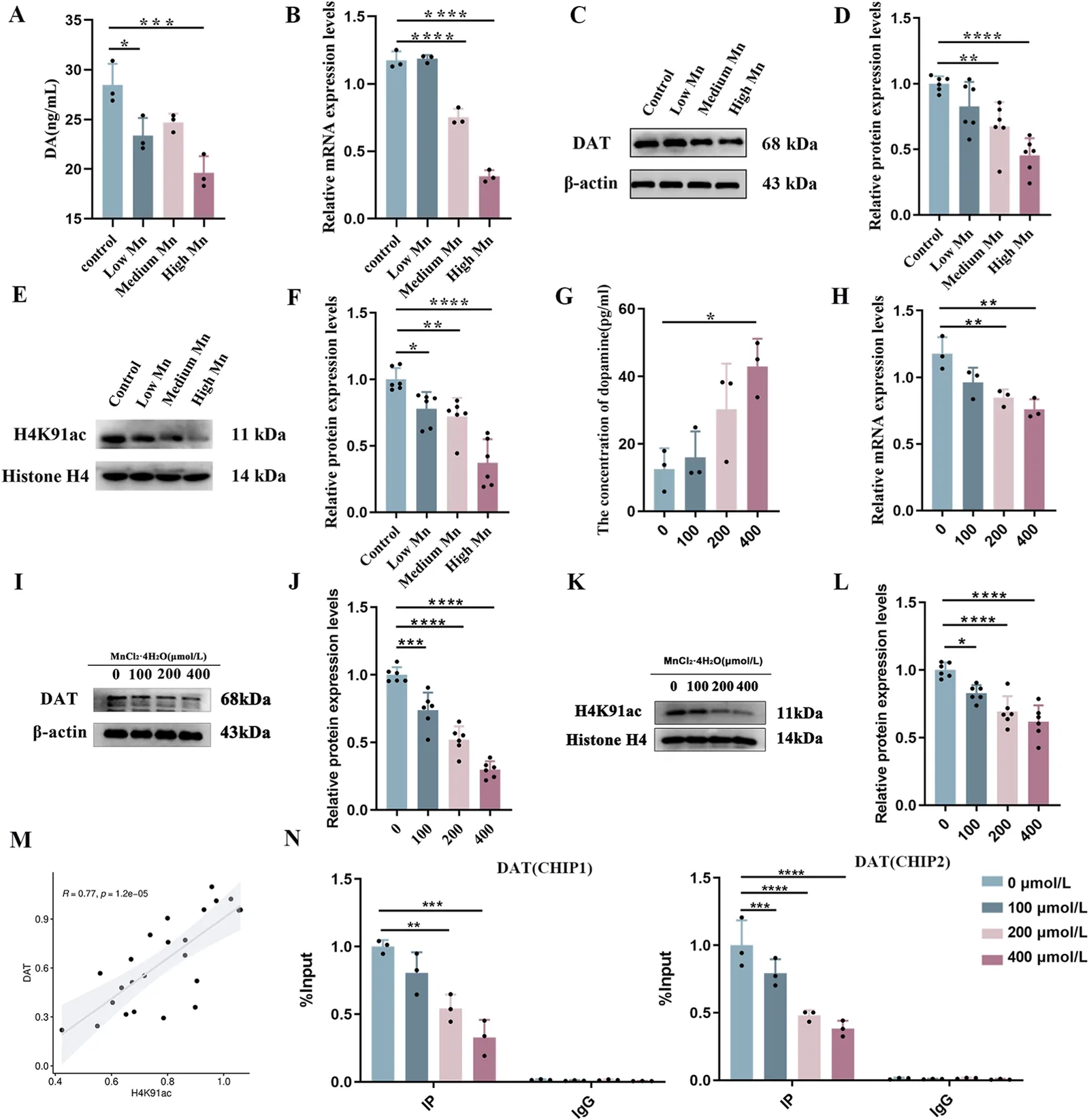
Manganese disrupts DA balance by reducing H4K91ac levels in the DAT promoter region. **(A)** LC-MS analysis of DA content in the striatum (n = 3). One-way ANOVA: F (3, 8) = 14.17, *p* = 0.0014. Post hoc Tukey’s test: Control vs. Low Mn, mean difference 95% CI [1.140, 9.060]; Control vs. High Mn, mean difference 95% CI [4.907, 12.83]. **(B)** RT-qPCR analysis of DAT mRNA levels in the striatum (n = 3). One-way ANOVA: F (3, 8) = 6.702, *p* < 0.0001. Post hoc Tukey’s test: Control vs. Medium Mn, mean difference 95% CI [0.298, 0.545]; Control vs. High Mn, mean difference 95% CI [0.737, 0.984]. **(C–F)** Western blot analysis of DAT and H4K91ac protein levels in the striatum and quantification (n = 6). One-way ANOVA: DAT, F (3, 20) = 14.41, *p* < 0.0001; H4K91ac, F (3, 20) = 21.91, *p* < 0.0001. Post hoc Tukey’s test: Control vs. Medium Mn, mean difference 95% CI [0.105, 0.545] (DAT); Control vs. High Mn, [0.328, 0.768] (DAT). Control vs. Low Mn, [0.021, 0.420] (H4K91ac); Control vs. Medium Mn, [0.081, 0.480]; Control vs. High Mn, [0.428, 0.826]. **(G)** ELISA analysis of DA content in cell supernatant (n = 3). One-way ANOVA: F (3, 8) = 6.735, *p* = 0.0140. Post hoc Tukey’s test: Control vs. High Mn, mean difference 95% CI [−0.333, −0.207]. **(H)** RT-qPCR analysis of DAT mRNA levels in cells (n = 3). One-way ANOVA: F (3, 8) = 10.62, *p* = 0.0037. Post hoc Tukey’s test: 0 μmol/L Mn vs. 200 μmol/L Mn, mean difference 95% CI [0.104, 0.556]; 0 μmol/L Mn vs. 400 μmol/L Mn, [0.192, 0.644]. **(I–L)** Western blot analysis of DAT and H4K91ac protein levels in cells and quantification (n = 6). One-way ANOVA: DAT, F (3, 20) = 64.41, *p* < 0.0001; H4K91ac, F (3, 20) = 19.72, *p* < 0.0001. Post hoc Tukey’s test: 0 μmol/L Mn vs. 100 μmol/L Mn, mean difference 95% CI [0.127, 0.395] (DAT); vs. 200 μmol/L, [0.345, 0.616]; vs. 400 μmol/L, [0.567, 0.836]. **(M)** Correlation analysis of H4K91ac and DAT protein expression. **(N)** ChIP-qPCR analysis of H4K91ac levels in the DAT promoter region (n = 3). One-way ANOVA: CHIP1, F (3, 8) = 19.75, *p* = 0.0005; CHIP2, F (3, 8) = 39.13, *p* < 0.0001. Post hoc Tukey’s test: 0 μmol/L Mn vs. 200 μmol/L, mean difference 95% CI [0.186, 0.727] (CHIP1); vs. 400 μmol/L, [0.402, 0.943] (CHIP1). 0 μmol/L vs. 100 μmol/L, [0.149, 0.507] (CHIP2); vs. 200 μmol/L, [0.441, 0.800]; vs. 400 μmol/L, [0.500, 0.858]. Data are expressed as mean ± SD. *p < 0.05, **p < 0.01, ***p < 0.001, ****p < 0.0001 vs. control group.

Manganese-induced neuronal damage is primarily mediated by abnormal DA neurotransmission in synapses. We first examined the expression of tyrosine hydroxylase (TH), a marker of dopaminergic neurons, and the results showed that excessive manganese exposure leads to damage to dopaminergic neurons (Supplementary Figures S1A–F). Next, we used LC–MS to measure DA levels and found that manganese exposure significantly reduced DA levels in the striatum (Figure 2A). Given the role of the DAT in DA homeostasis, we examined DAT expression and found that manganese inhibited DAT transcription and expression in the rat striatum (Figures 2B–D). We previously identified histone H4 lysine 91 acetylation (H4K91ac) as a differentially modified site in the striatum of rats with subchronic manganese poisoning (Ao et al., 2024). Consistent with this, manganese exposure decreased H4K91ac expression in the striatum (Figures 2E,F). In SH-SY5Y cells, manganese led to extracellular DA accumulation (Figure 2G) while inhibiting intracellular DAT transcription and protein levels, as well as H4K91ac levels (Figures 2H–L). Furthermore, DAT protein expression positively correlated with H4K91ac modification levels (Figure 2M). CHIP-qPCR analysis revealed that manganese reduced the level of H4K91ac modification in the DAT promoter region compared to the negative control IgG (Figure 2N). These results indicate that manganese may inhibits DAT transcription and expression by downregulating H4K91ac levels, leading to DA imbalance.

### 3.3 Manganese facilitates α-syn nuclear translocation, allowing its binding to histone H4

**FIGURE 3:**
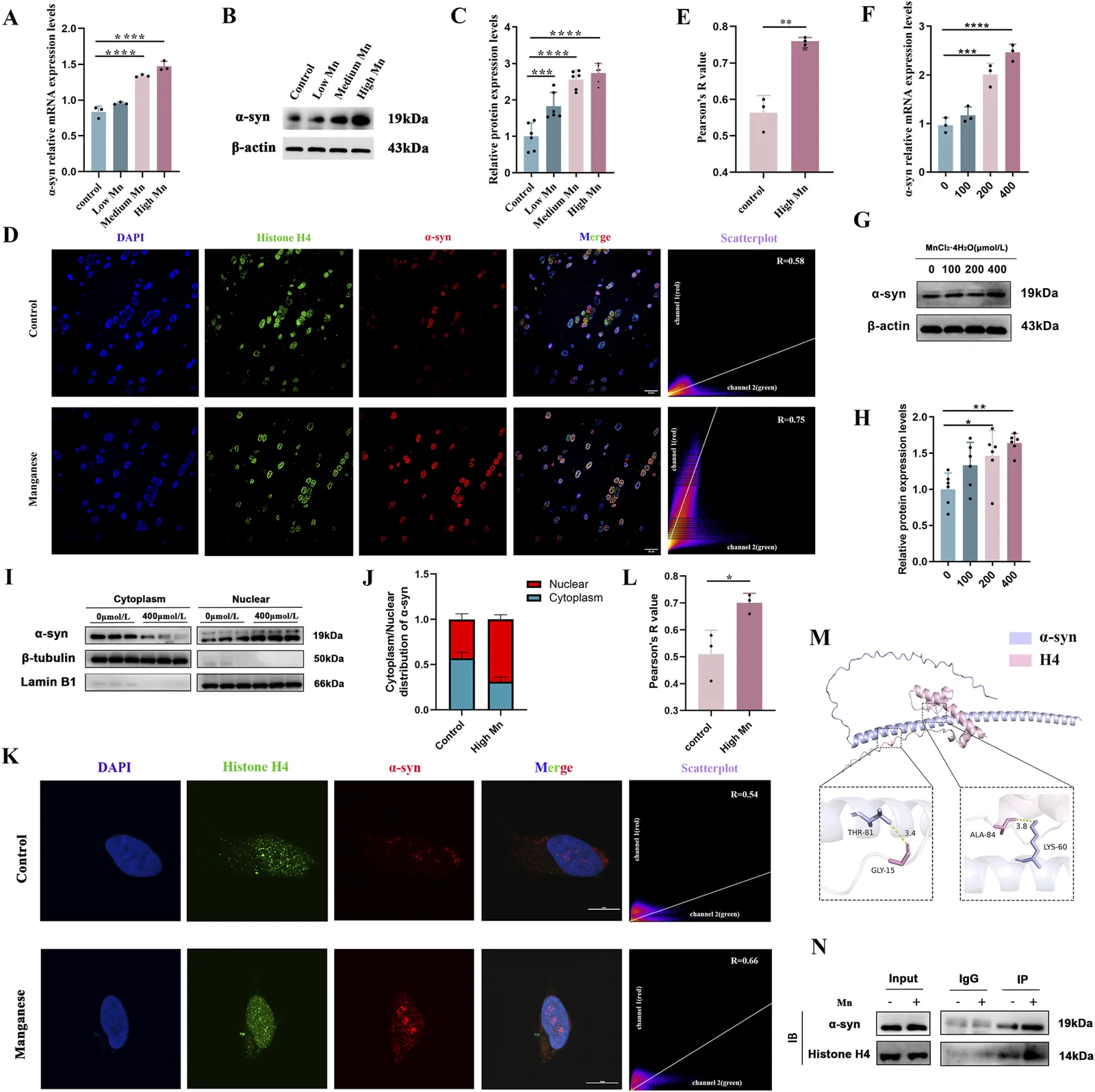
Manganese promotes α-syn nuclear translocation and its binding to histone H4. **(A)** RT-qPCR analysis of α-syn mRNA levels in the striatum (n = 3). One-way ANOVA: F (3, 8) = 96.08, *p* < 0.0001. Post hoc Tukey’s test: Control vs. Medium Mn, mean difference 95% CI [−0.630, −0.377]; Control vs. High Mn, [−0.764, −0.512]. **(B,C)** Western blot analysis of α-syn protein levels in the striatum and quantification (n = 6). One-way ANOVA: F (3, 20) = 36.17, *p* < 0.0001. Post hoc Tukey’s test: Control vs. Low Mn, mean difference 95% CI [−1.304, −0.357]; Control vs. Medium Mn, [−2.034, −1.088]; Control vs. High Mn, [−2.211, −1.264]. **(D,E)** Double immunofluorescence staining of α-syn and H4 in the striatum and colocalization coefficient quantification (n = 3), scale bar: 20 μm. Unpaired t-test: t (4) = 7.052, *p* = 0.0021. Control vs. High Mn, mean difference 95% CI [0.119, 0.274]. **(F)** RT-qPCR analysis of α-syn mRNA levels in cells (n = 3). One-way ANOVA: F (3, 8) = 44.69, *p* < 0.0001. Post hoc Tukey’s test: 0 μmol/L Mn vs. 200 μmol/L Mn, mean difference 95% CI [−1.472, −0.615]; vs. 400 μmol/L, [−1.924, −1.067]. **(G,H)** Western blot analysis of α-syn protein levels in cells and quantification (n = 6). One-way ANOVA: F (3, 20) = 5.931, *p* = 0.0046. Post hoc Tukey’s test: 0 μmol/L Mn vs. 200 μmol/L, mean difference 95% CI [−0.858, −0.064]; vs. 400 μmol/L, [−1.035, −0.240]. **(I,J)** Western blot analysis of α-syn expression in cytoplasm and nucleus, and quantification (n = 3). Unpaired t-test: cytoplasm, t (4) = 5.575, p = 0.0051; nucleus, t (4) = 5.575, *p* = 0.0051. 0 μmol/L vs. 400 μmol/L: mean difference 95% CI [−0.726, −0.401] (cytoplasm) and [0.012, 0.574] (nucleus). **(K,L)** Double immunofluorescence staining of H4 and α-syn in cells and colocalization coefficient quantification (n = 3), scale bar: 10 μm. Unpaired t-test: t (4) = 3.431, *p* = 0.0265. Control vs. Mn, mean difference 95% CI [0.036, 0.344]. **(M)** Molecular docking diagram of α-syn and H4. **(N)** Co-immunoprecipitation analysis of α-syn and H4 in nuclei of manganese-treated cells. Data are expressed as mean ± SD. *p < 0.05, **p < 0.01, ***p < 0.001, ****p < 0.0001 vs. control group.

Manganese promotes α-syn overaccumulation in the striatum and cells (Figures 3A–C,F–H), consistent with reports that the overaccumulation of α-syn leads to neurotoxicity, and elevated α-syn levels reduce histone H3 acetylation (Kontopoulos et al., 2006). Immunofluorescence revealed that manganese also promotes α-syn nuclear translocation and increases its colocalization with histone H4 in the nucleus (Figures 3D,E,K,L). Subcellular fractionation confirmed that manganese induces excessive α-syn accumulation in the nucleus (Figures 3I,J). Molecular docking suggested a direct interaction between α-syn and H4, with hydrogen bonds forming between THR-81 and LYS-60 of α-synuclein and GLY-15 and ALA-84 of H4 (Figure 3M). Co-immunoprecipitation further confirmed that manganese promotes the binding of α-syn to H4 in the nucleus (Figure 3N). These findings indicate that manganese promotes α-syn overexpression, enabling its nuclear translocation and binding to histone H4.

### 3.4 Reducing α-syn increases H4K91ac levels and enhances DAT expression

**FIGURE 4:**
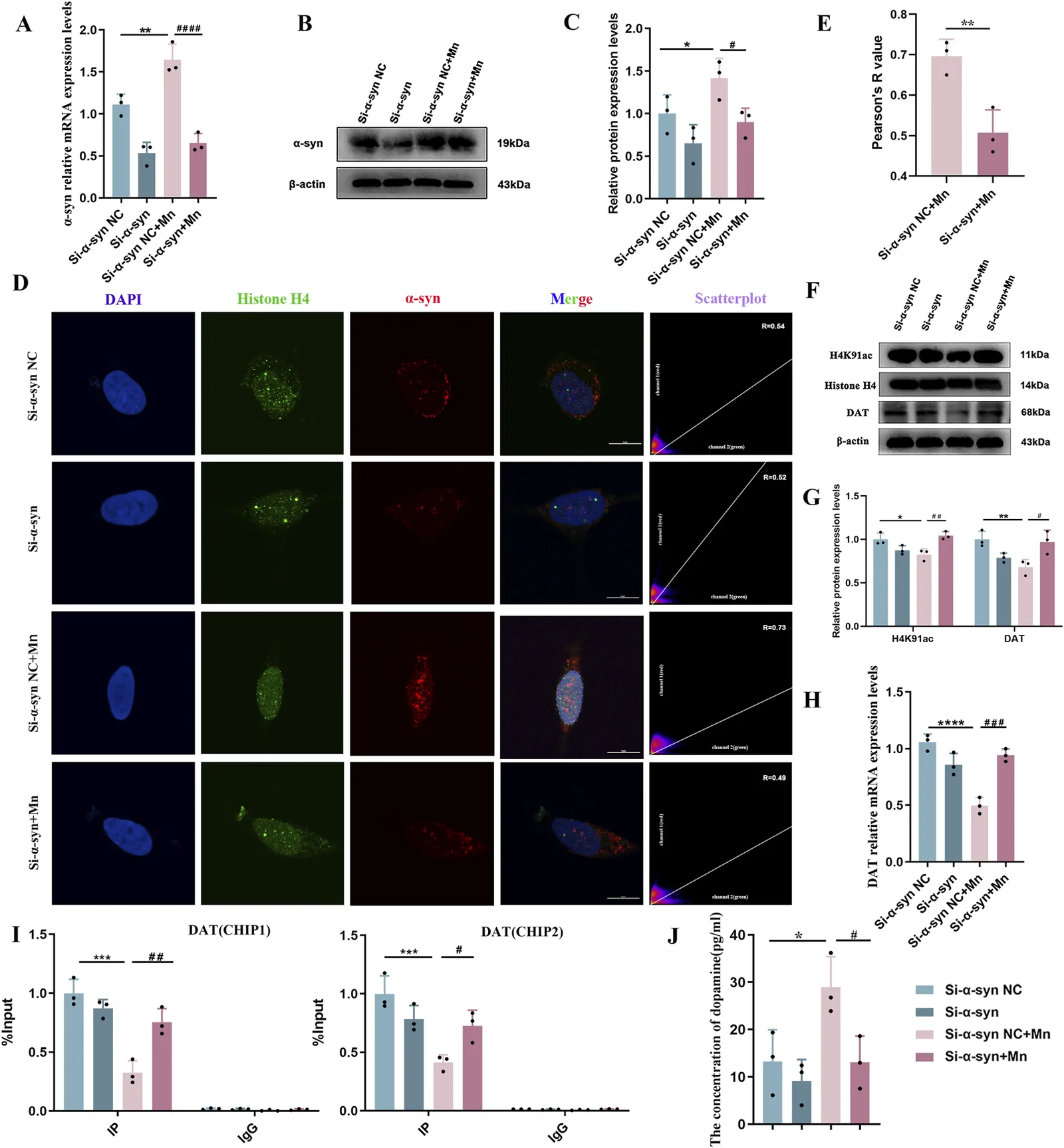
Knockdown of α-syn upregulates H4K91ac levels and promotes DAT expression. **(A)** mRNA levels of α-syn in manganese-treated cells after α-syn knockdown (n = 3). One-way ANOVA: F (3, 8) = 38.30, *p* < 0.0001. Post hoc Tukey’s test: Si-α-syn NC vs. Si-α-syn NC + Mn, mean difference 95% CI [0.202, 0.867]; Si-α-syn NC + Mn vs. Si-α-syn + Mn, [0.659, 1.324]. **(B,C)** Protein levels of α-synuclein in manganese-treated cells after α-syn knockdown and quantification (n = 3). One-way ANOVA: F (3, 8) = 6.940, *p* = 0.0129. Post hoc Tukey’s test: Si-α-syn NC vs. Si-α-syn NC + Mn, mean difference 95% CI [0.058, 0.939]; Si-α-syn NC + Mn vs. Si-α-syn + Mn, [0.024, 0.904]. **(D,E)** Double immunofluorescence staining images of α-syn and histone H4 in manganese-treated cells after α-syn knockdown and quantification of the colocalization coefficient between the two (n = 3), scale bar: 10 μm. Unpaired t-test: t (4) = 4.670, *p* = 0.0095. Si-α-syn NC + Mn vs. Si-α-syn + Mn, mean difference 95% CI [−0.303, −0.077]. **(F,G)** Protein levels of H4K91ac and DAT in manganese-treated cells after α-syn knockdown and quantification (n = 3). One-way ANOVA: DAT, F (3, 8) = 7.452, *p* = 0.0105; H4K91ac, F (3, 8) = 8.896, *p* = 0.0063. Post hoc Tukey’s test: Si-α-syn NC vs. Si-α-syn NC + Mn, [−0.546, −0.093] (DAT); Si-α-syn NC + Mn vs. Si-α-syn + Mn, [−0.518, −0.065] (DAT). Si-α-syn NC vs. Si-α-syn NC + Mn, [−0.317, −0.035] (H4K91ac); Si-α-syn NC + Mn vs. Si-α-syn + Mn, [−0.360, −0.078] (H4K91ac). **(H)** mRNA levels of *DAT* in manganese-treated cells after α-syn knockdown (n = 3). One-way ANOVA: F (3, 8) = 30.42, *p* = 0.0001. Post hoc Tukey’s test: Si-α-syn NC vs. Si-α-syn NC + Mn, mean difference 95% CI [−0.740, −0.382]; Si-α-syn NC + Mn vs. Si-α-syn + Mn, [−0.624, −0.266]. **(I)** Effect of α-syn knockdown on H4K91ac levels in the *DAT* promoter region in manganese-treated cells (n = 3). One-way ANOVA: CHIP1, F (3, 8) = 23.64, *p* = 0.0002; CHIP2, F (3, 8) = 12.06, *p* = 0.0024. Post hoc Tukey’s test: Si-α-syn NC vs. Si-α-syn NC + Mn, [−0.921, −0.430] (CHIP1); Si-α-syn NC + Mn vs. Si-α-syn + Mn, [−0.674, −0.183] (CHIP1). Si-α-syn NC vs. Si-α-syn NC + Mn, [−0.873, −0.303] (CHIP2); Si-α-syn NC + Mn vs. Si-α-syn + Mn, [−0.601, −0.031] (CHIP2). **(J)** Changes in DA content in cell supernatant (n = 3). One-way ANOVA: F (3, 8) = 6.748, *p* = 0.0139. Post hoc Tukey’s test: Si-α-syn NC vs. Si-α-syn NC + Mn, mean difference 95% CI [1.936, 29.43]; Si-α-syn NC + Mn vs. Si-α-syn + Mn, [2.141, 29.64]. Data are expressed as mean ± SD. *p < 0.05, **p < 0.01, ***p < 0.001, ****p < 0.0001 vs. control group. #*p* < 0.05, ##*p* < 0.01, ###*p* < 0.001, ####*p* < 0.0001 vs. Si-α-syn NC + Mn group.

To investigate α-syn’s role in regulating DAT expression via H4K91ac in the nucleus, we used siRNA to knock down α-syn (Figures 4A–C). This significantly reduced α-syn’s co-localization with histone H4 in the nucleus (Figures 4D,E). Western blot analysis revealed that α-syn downregulation upregulated H4K91ac levels, promoting DAT transcription and expression (Figures 4F–H). CHIP-qPCR confirmed that α-syn downregulation increased H4K91ac enrichment in the *DAT* promoter region (CHIP1, CHIP2) (Figure 4I). Furthermore, downregulation of α-syn significantly decreased extracellular DA content (Figure 4J). These findings suggest that manganese-induced α-syn overexpression increases its nuclear translocation and binding to histone H4, reducing H4K91ac levels, inhibiting DAT transcription and expression, and ultimately disrupting DA balance.

### 3.5 Manganese induces downregulation of H4K91ac via the Trim28/α-syn axis

**FIGURE 5:**
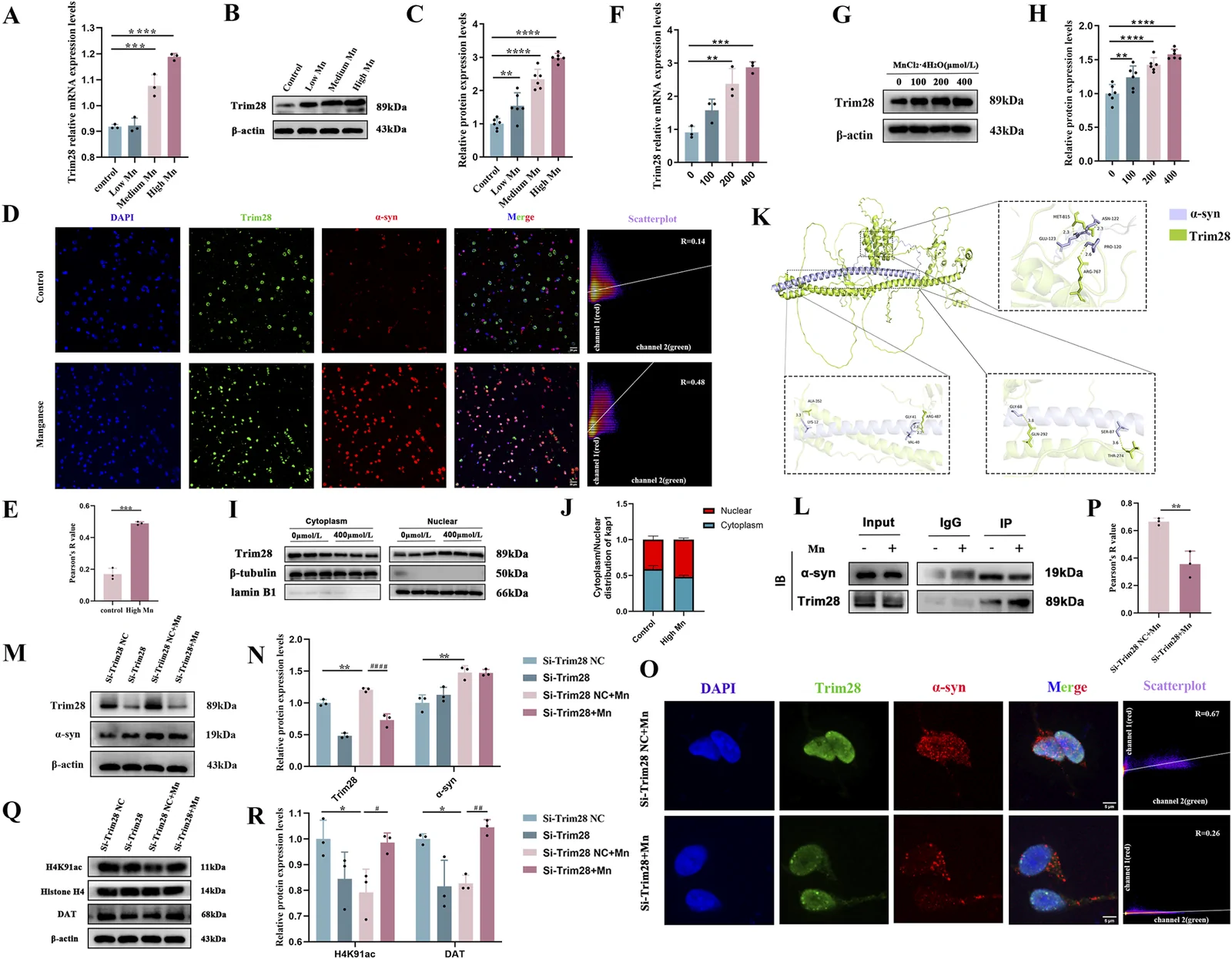
Manganese mediates α-syn nuclear translocation via Trim28. **(A)** RT-qPCR assay of *Trim28* mRNA levels in the striatum (n = 3). One-way ANOVA: F (3, 8) = 71.05, *p* < 0.0001. Post hoc Tukey’s test: Control vs. Medium Mn, mean difference 95% CI [−0.221, −0.095]; Control vs. High Mn, [−0.333, −0.207]. **(B,C)** WB assay of Trim28 protein levels in the striatum and quantitative analysis (n = 6). One-way ANOVA: F (3, 20) = 64.03, *p* < 0.0001. Post hoc Tukey’s test: Control vs. Low Mn, mean difference 95% CI [−0.939, −0.151]; Control vs. Medium Mn, [−1.731, −0.947]; Control vs. High Mn, [−2.377, −1.592]. **(D,E)** Double immunofluorescence staining images of Trim28 and α-syn in the striatum and quantitative analysis of their co-localization coefficient (n = 3), scale bar: 20 μm. Unpaired t-test: t (4) = 14.81, *p* = 0.0001. Control vs. High Mn, mean difference 95% CI [0.260, 0.380]. **(F)** RT-qPCR assay of *Trim28* mRNA levels in cells (n = 3). One-way ANOVA: F (3, 8) = 23.44, *p* = 0.0003. Post hoc Tukey’s test: 0 μmol/L Mn vs. 200 μmol/L, mean difference 95% CI [−2.198, −0.738]; vs. 400 μmol/L, [−2.695, −1.236]. **(G,H)** WB assay of Trim28 protein levels in cells and quantitative analysis (n = 6). One-way ANOVA: F (3, 20) = 24.30, *p* < 0.0001. Post hoc Tukey’s test: 0 μmol/L vs. 100 μmol/L, mean difference 95% CI [−0.422, −0.058]; vs. 200 μmol/L, [−0.607, −0.243]; vs. 400 μmol/L, [−0.762, −0.398]. **(I,J)** WB assay of Trim28 expression in cytoplasm and nucleus, and quantitative analysis (n = 3). Unpaired t-test: cytoplasm, t (4) = 3.353, *p* = 0.0285; nucleus, t (4) = 3.353, *p* = 0.0285. 0 μmol/L vs. 400 μmol/L: mean difference 95% CI [−0.277, −0.106] (cytoplasm) and [0.148, 0.339] (nucleus). **(K)** Molecular docking diagram of Trim28 and α-syn. **(L)** Co-immunoprecipitation analysis of Trim28 and α-syn after manganese treatment. **(M,N)** Trim28, α-syn protein levels and quantitative analysis in manganese-treated cells after Trim28 knockdown (n = 3). One-way ANOVA: Trim28, F (3, 8) = 80.89, p < 0.0001; α-syn, F (3, 8) = 16.50, *p* = 0.0009. Post hoc Tukey’s test: Si-Trim28 NC vs. Si-Trim28 NC + Mn, [0.064, 0.350] (Trim28); Si-Trim28 NC + Mn vs. Si-Trim28 + Mn, [0.336, 0.622] (Trim28). Si-Trim28 NC vs. Si-Trim28 NC + Mn, [0.236, 0.724] (α-syn). **(O,P)** Double immunofluorescence staining images of Trim28 and α-syn in manganese-treated cells after Trim28 knockdown and quantitative analysis of their co-localization coefficient (n = 3), scale bar: 5 μm. Unpaired t-test: t (4) = 5.461, *p* = 0.0055. Si-Trim28 NC + Mn vs. Si-Trim28 + Mn, mean difference 95% CI [−0.468, −0.152]. **(Q,R)** H4K91ac, DAT protein levels and quantitative analysis in manganese-treated cells after Trim28 knockdown (n = 3). One-way ANOVA: H4K91ac, F (3, 8) = 4.943, *p* = 0.0315; DAT, F (3, 8) = 13.11, *p* = 0.0019. Post hoc Tukey’s test: Si-Trim28 NC vs. Si-Trim28 NC + Mn, [−0.398, −0.019] (H4K91ac); Si-Trim28 NC + Mn vs. Si-Trim28 + Mn, [−0.384, −0.005] (H4K91ac). Si-Trim28 NC vs. Si-Trim28 NC + Mn, [−0.305, −0.040] (DAT); Si-Trim28 NC + Mn vs. Si-Trim28 + Mn, [−0.351, −0.086] (DAT). Data are expressed as mean ± SD. *p < 0.05, **p < 0.01, ***p < 0.001, ****p < 0.0001 vs. control group. #*p* < 0.05, ##*p* < 0.01, ###*p* < 0.001, ####*p* < 0.0001 vs. Si-Trim28 NC + Mn group.

Trim28 is a key partner in the nuclear accumulation of α-syn, but its molecular mechanism in manganese-induced neurotoxicity remains unclear. We found that manganese treatment upregulates Trim28 transcription and protein expression in the striatum and cells (Figures 5A–C,F–H). Immunofluorescence showed increased Trim28 and α-syn co-localization in these areas (Figures 5D,E; Supplementary Figure S3A), with increased Trim28 expression in the nucleus (Figures 5I,J). As shown in Figure 5K, molecular docking suggested a strong binding affinity between Trim28 and α-syn, supported by eight hydrogen bond interactions (e.g., Trim28 ALA-353 and α-syn LYS-12). Co-IP confirmed enhanced interaction between Trim28 and α-syn after manganese treatment (Figure 5L). Knockdown of Trim28 via siRNA (Figures 5M,N) did not affect total α-syn protein expression but reduced α-syn nuclear translocation (Figures 5O,P) and upregulated H4K91ac and DAT expression (Figures 5Q,R). These results suggest that manganese promotes the nuclear translocation of α-syn via Trim28, inhibiting H4K91ac, which affects DAT expression and leads to DA imbalance.

### 3.6 Network pharmacology analysis

**FIGURE 6:**
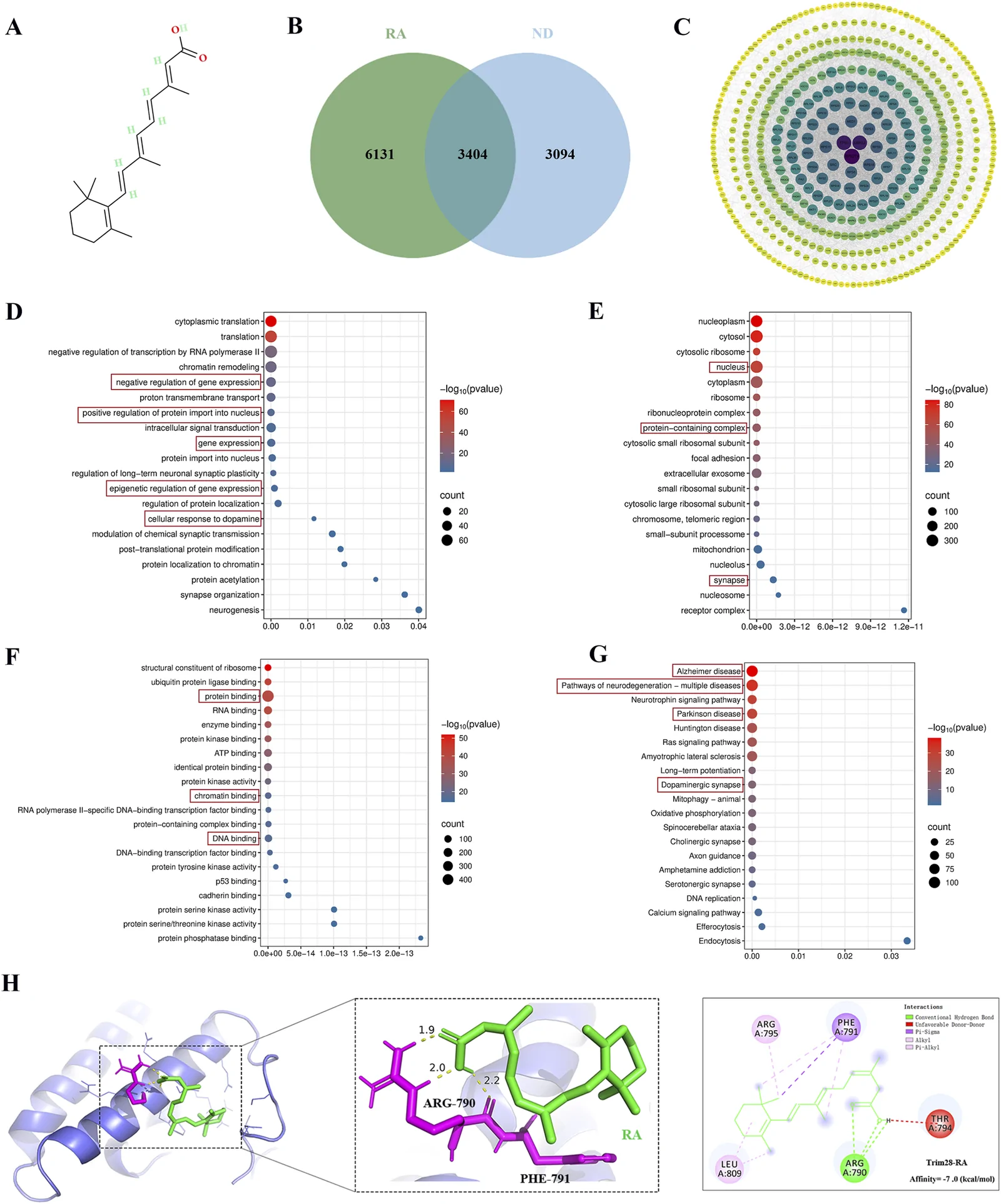
Network pharmacology analysis. **(A)** Chemical structure of retinoic acid. **(B)** Venn diagram of retinoic acid (RA) and neurodegenerative disease (ND) related targets. **(C)** Protein-protein interaction network diagram. **(D)** GO enrichment analysis: biological process. **(E)** GO enrichment analysis: cellular component. **(F)** GO enrichment analysis: molecular function. **(G)** KEGG enrichment analysis. **(H)** Molecular docking of retinoic acid and Trim28.

To investigate Trim28 as a potential therapeutic target for retinoic acid intervention in neurodegenerative diseases, we performed network pharmacology analysis. We identified 3404 common targets between retinoic acid (9535 targets from CTD and Swiss Target Prediction databases, chemical structure in Figure 6A) and neurodegenerative diseases (6498 targets from GeneCards and OMIM databases, Venn diagram in Figure 6B). A protein-protein interaction (PPI) network of the top 500 genes (based on Degree value) was constructed using networkanalyst and visualized in Cytoscape (confidence level 0.9, Figure 6C). Trim28 emerged as a key target.

To clarify retinoic acid’s mechanism in neurodegenerative diseases, GO enrichment analysis of 500 targets revealed enrichment in gene expression and protein localization processes (e.g., negative regulation of gene expression, positive regulation of protein import into the nucleus, epigenetic regulation of gene expression), cellular components (nucleoplasm, nucleus, protein-containing complex, synapse), and molecular functions (protein, chromatin, and DNA binding) (Figures 6D–F). KEGG pathway analysis highlighted neurodegenerative disease pathways (Alzheimer’s, Parkinson’s, etc.) and synapse-related pathways (dopaminergic, cholinergic, serotonergic) (Figure 6G). Molecular docking results indicate that there is a theoretical binding affinity between retinoic acid and the bromodomain of Trim28 (−7 kcal/mol, 3 hydrogen bonds) (Figure 6H).

### 3.7 Retinoic acid repairs manganese-induced damage in SH-SY5Y cells by modulating Trim28

**FIGURE 7:**
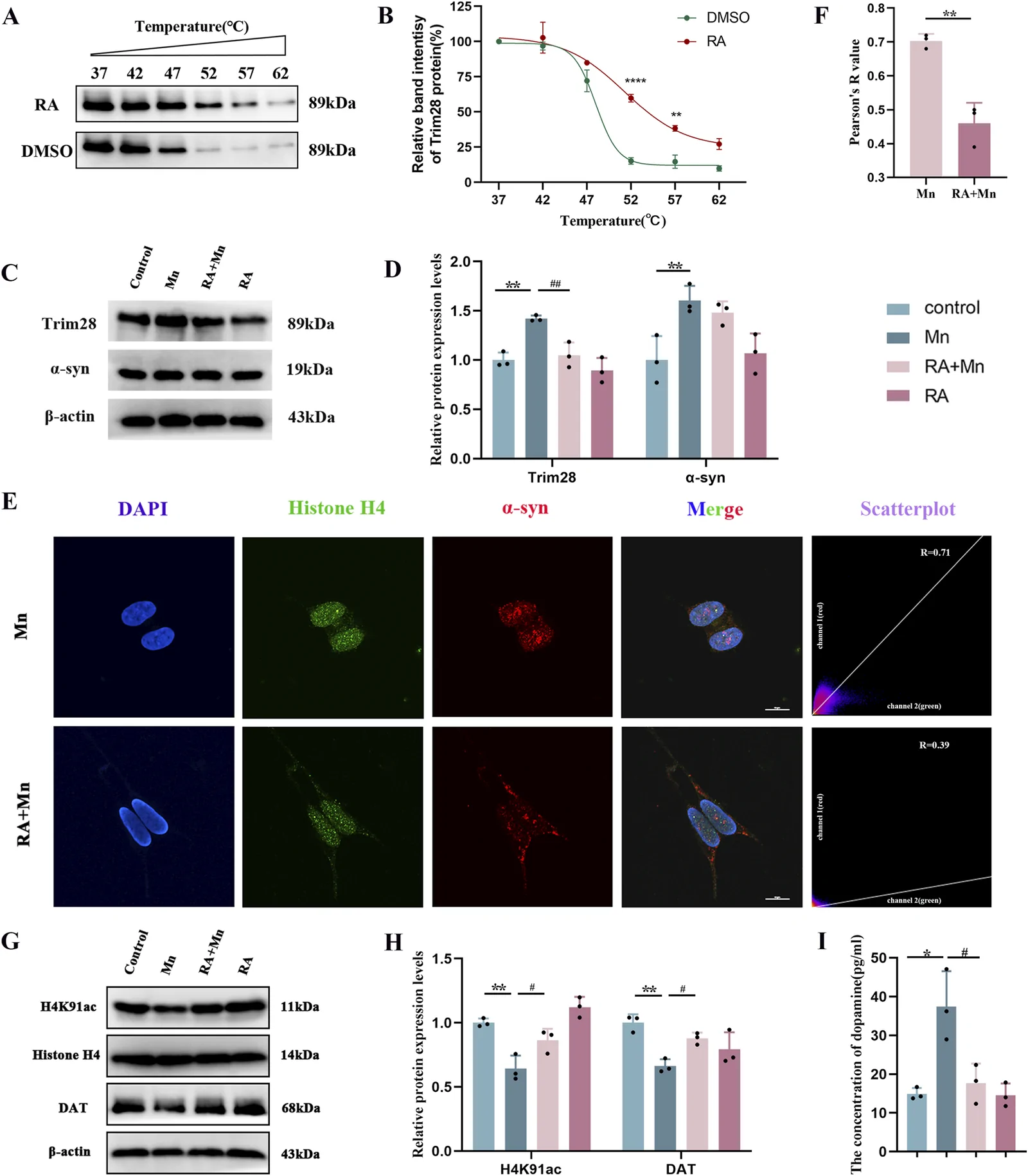
Retinoic acid restores manganese-induced damage to SH-SY5Y cells by regulating Trim28. **(A,B)** CETSA analysis between RA and Trim28 (n = 3). Two-way ANOVA with Sidak’s multiple comparisons test: at 50 °C, *p* < 0.0001; at 57 °C, *p* = 0.0072. **(C,D)** Western blot analysis of Trim28 and α-syn protein levels and quantification (n = 3). One-way ANOVA: Trim28, F (3, 8) = 16.00, *p* = 0.0010; α-syn, F (3, 8) = 8.039, *p* = 0.0085. Post hoc Tukey’s test: Control vs. Mn, mean difference 95% CI [−0.682, −0.161] (Trim28) and [-1.084, −0.127] (α-syn); Mn vs. RA + Mn, [0.112, 0.633] (Trim28) and [−0.353, 0.604] (α-syn). **(E,F)** Double immunofluorescence staining images of H4 and α-syn in cells and quantification of their co-localization coefficient (n = 3), scale bar: 10 μm. Unpaired t-test: t (4) = 6.556, *p* = 0.0028. Mn vs. RA + Mn, mean difference 95% CI [−0.346, −0.140]. **(G,H)** Western blot analysis of H4K91ac and DAT protein levels and quantification (n = 3). One-way ANOVA: H4K91ac, F (3, 8) = 19.62, *p* = 0.0005; DAT, F (3, 8) = 9.043, *p* = 0.0060. Post hoc Tukey’s test: Control vs. Mn, mean difference 95% CI [0.148, 0.569] (H4K91ac) and [0.124, 0.552] (DAT); Mn vs. RA + Mn, [−0.431, −0.010] (H4K91ac) and [−0.345, −0.084] (DAT). **(I)** Changes in DA content in cell supernatant (n = 3). One-way ANOVA: F (3, 8) = 11.95, *p* = 0.0025. Post hoc Tukey’s test: Control vs. Mn, mean difference 95% CI [9.613, 35.40]; Mn vs. RA + Mn, [6.855, 32.64]. Data are expressed as mean ± SD. *p < 0.05, **p < 0.01, ***p < 0.001, ****p < 0.0001 vs. control group. #*p* < 0.05, ##*p* < 0.01, ###*p* < 0.001, ####*p* < 0.0001 vs. Mn group.

Based on network pharmacology and molecular docking analyses, Trim28 may be a potential target for retinoic acid. We subsequently performed a CETSA analysis, which revealed that retinoic acid significantly enhanced the thermal stability of Trim28, confirming the existence of an interaction between retinoic acid and Trim28 (Figures 7A,B). Furthermore, Trim28 protein levels were significantly reduced in retinoic acid-treated cells, whilst the expression of α-syn protein remained unaffected (Figures 7C,D). Retinoic acid treatment also decreased α-syn accumulation and its co-localization with histone H4 in the nucleus (Figures 7E,F), while upregulating H4K91ac and DAT protein levels (Figures 7G,H). Furthermore, retinoic acid improved manganese-induced extracellular DA accumulation (Figure 7I). These findings suggest that retinoic acid suppresses the nuclear translocation of α-syn by modulating Trim28, thereby restoring the DA homeostasis disrupted by manganese exposure in SH-SY5Y cells.

## 4 Discussion

Manganese is a common element found in soil, water, and rocks in inorganic forms, while organic manganese compounds are used in steel production, pesticides, and gasoline additives (Studer et al., 2022). Although essential in small amounts, excessive manganese intake is toxic, particularly to the central nervous system (Wu et al., 2022). Overexposure can cause manganism, a condition resembling Parkinson’s disease, with symptoms such as bradykinesia, rigidity, and tremor, severely impacting quality of life. Studies of foundry workers revealed a high prevalence of Parkinsonism (42.2%) linked to chronic manganese exposure (Rohani et al., 2022). Animal studies further demonstrate that excessive manganese leads to accumulation in tissues like the hippocampus, serum, teeth, and hair, impairing learning and memory (Liang et al., 2016). Our research also indicates manganese accumulation in the striatum, affecting learning, memory, and motor functions. Despite growing awareness of environmental manganese pollution, the precise mechanisms of its toxicity remain unclear.

DA is synthesised in the midbrain dopaminergic neurons, which projects to the striatum, hypothalamus, and cortex, regulating movement and motivation via DA release (Gentry et al., 2019). Degeneration of these neurons reduces striatal DA, causing Parkinson’s disease (PD) symptoms like bradykinesia and tremor (Ohashi et al., 2006). Conversely, aconitine increases synaptic cleft DA, inducing SH-SY5Y cell toxicity (Zhou et al., 2022). Our findings align with these studies, showing that manganese exposure disrupts DA homeostasis by reducing striatal DA content while increasing extracellular DA levels. This study found that manganese exposure has seemingly contradictory effects on DA homeostasis under *in vivo* and *in vitro* conditions: following 18 weeks of manganese exposure, total DA content in the striatum of rats was significantly reduced; whereas *in vitro* in SH-SY5Y cells, extracellular DA levels increased following manganese exposure. We believe that these results are not contradictory, but may instead reflect different manifestations of manganese-induced dopaminergic dysfunction at different time points. The reduction in striatal DA *in vivo* may reflect a loss of dopaminergic synaptic terminal function or inhibition of tyrosine hydroxylase activity resulting from chronic manganese exposure, indicating a decline in overall DA synthesis and storage capacity (Stanwood et al., 2009; Guilarte, 2010). By contrast, the elevated extracellular DA levels *in vitro* may stem from impaired DAT reuptake function during early manganese exposure, leading to abnormal accumulation of extracellular DA (Roth et al., 2013); this dysfunction may occur prior to neuronal loss (Guilarte et al., 2008). Consequently, *in vivo* models of chronic exposure may reflect a terminal or near-terminal state of DA depletion, whilst *in vitro* models may capture the accumulation of extracellular DA during the early compensatory-to-imbalanced transition phase.

Imbalances in DA homeostasis, often involving DAT dysfunction, are implicated in PD, bipolar disorder, schizophrenia, ADHD, and other neurological disorders (Rastedt et al., 2017). DAT, a key regulator of synaptic cleft DA, reuptakes DA into presynaptic neurons. DAT dysfunction-induced DA homeostasis imbalance is considered a potential cause of various diseases (Hovde et al., 2019). We hypothesize that manganese-induced DA homeostasis imbalance is related to DAT dysfunction. This study confirms that manganese inhibits DAT expression, leading to DA homeostasis imbalance, consistent with findings that DAT deletion alters DA levels and signaling, mediating disease (Leo et al., 2018). Therefore, elucidating the epigenetic regulatory mechanisms underlying changes in DAT expression is crucial for understanding the molecular basis of manganese neurotoxicity.

Histone acetylation, an epigenetic modification influencing gene expression, is implicated in the neurodegenerative processes of neurodegenerative diseases (Rossaert et al., 2019; Lin et al., 2023). We previously observed a significant reduction in H4K91ac in the striatum of manganese-exposed rats. Investigating the potential of H4K91ac to regulate DAT transcriptional expression, CHIP-qPCR revealed that manganese exposure reduces H4K91ac levels in the DAT promoter region. This suggests that manganese may regulate the transcriptional expression of the DAT via H4K91ac, thereby disrupting DA homeostasis. This highlights the role of histone acetylation in manganese-induced neurotoxicity. Regarding the relationship between H4K91ac and DAT transcriptional regulation, current evidence from this study is limited to correlation and ChIP-qPCR enrichment analyses. Establishing causality necessitates direct modulation of H4K91ac levels, such as via overexpression/knockout of H4K91 site-specific acetyltransferases or the use of H4K91R mutants, followed by observation of corresponding changes in DAT transcriptional expression. Under the current data, we define H4K91ac as a potential epigenetic biomarker for DAT transcriptional status. The disruption of histone acetylation balance is linked to disease. For example, postmortem PD patients exhibit altered H3K9ac, H3K14ac, and H3K18ac levels in the motor cortex compared to non-PD patients (Gebremedhin and Rademacher, 2016). Furthermore, H3K9ac, H3K14ac, H4K5ac, and H4K12ac levels are reduced in the hippocampal CA3 region of mice with cognitive impairment (Feng et al., 2017). Consistent with these findings, our study shows that manganese exposure inhibits H4K91ac levels. Combined with prior observations that manganese exposure reduces H3K27ac and H3K36ac levels (Liu et al., 2024b; Liu et al., 2024a), this indicates that manganese exposure disrupts histone acetylation balance, though the underlying molecular mechanism requires further investigation.

Histone acetylation, a key factor in disease development, is dynamically regulated by histone acetyltransferases (HATs) and histone deacetylases (HDACs). Determining the cause of acetylation imbalances is challenging due to the multi-site activity of these enzymes (Wang et al., 2023). Studies in paraquat-treated mice revealed α-syn co-localization with histones in the nucleus accumbens of nigral neurons (Goers et al., 2003). Our study found that manganese exposure similarly increased α-syn and histone H4 co-localization in dopaminergic neuron nuclei. α-syn overexpression can also alter H3 acetylation and inhibit DNA repair gene expression (Paiva et al., 2017). We hypothesized that α-syn translocates to the nucleus, binds to histones, and inhibits histone acetylation, thereby downregulating gene transcription and promoting disease. Our findings indicate that manganese exposure enhances α-syn binding to histone H4, and α-syn downregulation reverses manganese-induced reductions in H4K91ac and DAT expression, restoring DA homeostasis. We demonstrated that manganese promotes α-syn overaccumulation and nuclear translocation, leading to α-syn binding to H4, which hinders H4 modification, reduces H4K91ac levels, and inhibits DAT transcript expression. The mechanism of α-syn nuclear translocation, however, remains unclear.

Classical nuclear protein import relies on importin α/β recognizing a nuclear localization signal (NLS) on the target protein for transport through the nuclear pore complex (NPC) (Yang et al., 2023). α-syn lacks a classical NLS (Pinho et al., 2019), and while some studies suggest importin α involvement, a direct interaction remains unconfirmed (Ma et al., 2014). Given that Trim28, a transcription regulator containing an NLS, enters the nucleus via the import proteins α/β (Moriyama et al., 2015), does α-syn enter the nucleus by binding to Trim28? Previous studies have shown that Trim28 promotes the accumulation of α-syn in the cell nucleus (Rousseaux et al., 2016). Our study also found that manganese exposure enhances the binding of α-syn to Trim28, whilst downregulation of Trim28 reduces the translocation of α-syn into the nucleus, further confirming that Trim28 acts as a molecular chaperone for the nuclear transport of α-syn. So, how does Trim28 facilitate the nuclear import of α-synuclein? We hypothesise that there are several possible mechanisms: firstly, Trim28 itself contains a classic NLS sequence; upon binding to α-synuclein, it may act as a ‘hitchhiker’, transporting the latter into the nucleus. Secondly, Trim28 may interact with nuclear pore complex proteins, causing α-synuclein to accumulate near the nuclear pore and thereby facilitating its nuclear import.

Notably, Trim28 downregulation altered α-syn distribution without affecting its expression, suggesting that altered localization impacts its function and potentially contributes to disease. We further demonstrate that manganese-induced dysregulation of histone acetylation and DA homeostasis are partly caused by the Trim28/α-syn axis. Regarding how the α-synuclein/Trim28 complex reduces H4K91ac levels upon nuclear entry, we hypothesise that two mechanisms may be at play: firstly, we have observed that α-synuclein binds to the core histone H4. This interaction may physically impede the accessibility of the catalytic domain of histone acetyltransferases to this site. Furthermore, Trim28, acting as a scaffold protein, is known to recruit the histone deacetylase complex NuRD (Srinivasan et al., 2022). He binding of α-synuclein could potentially enhance Trim28’s affinity for such complexes, thereby actively deacetylating H4K91. We will conduct further research into this hypothesis in the future.

Considering the lack of effective treatments for manganese toxicity and the unknown target of retinoic acid in neurodegenerative diseases, we employed network pharmacology analysis and molecular docking to explore potential targets for retinoic acid. These analyses suggested that Trim28 may be a potential binding protein for retinoic acid. In the SH-SY5Y cell model, we found that retinoic acid treatment reduced Trim28 protein levels, accompanied by a partial recovery of H4K91ac and DAT expression, and improved manganese-induced DA imbalance. Furthermore, we found that retinoic acid did not affect the expression levels of α-syn, but altered its distribution between the nucleus and the cytoplasm. These results suggest that, at the cellular level, retinoic acid may influence the nuclear translocation of α-syn via Trim28, thereby regulating downstream epigenetic and transcriptional events. Retinoic acid can induce neuronal differentiation, leading to the extension of neurites and increased expression of markers of mature neurons. This may be one of the reasons for the observed increase in DAT. However, we found that retinoic acid treatment reduced the nuclear localisation of α-synuclein, which is not a classic marker of differentiation; this supports the existence of a component unrelated to differentiation.

The molecular mechanisms by which retinoic acid regulates Trim28 and epigenetics may be achieved through two pathways. Firstly, this could be mediated by the classical RAR/RXR pathway. Retinoic acid, as an endogenous ligand for RARα/β/γ and RXR, enters the cell and binds to nuclear receptors, inducing conformational changes in the receptors (Chambon, 1996). The TRIM family, known as general transcriptional co-regulators, has been shown to be recruited to ligand-bound nuclear receptor complexes (Herquel et al., 2011). Therefore, the observed changes in Trim28 levels following retinoic acid treatment may reflect indirect regulation of Trim28 expression or stability subsequent to RAR/RXR signaling pathway activation. Secondly, retinoic acid may directly bind to Trim28. Our molecular docking analysis suggests that retinoic acid theoretically possesses affinity for Trim28. We further confirmed an interaction between retinoic acid and Trim28 through CETSA. However, CETSA signals can arise from direct binding or ligand-induced indirect conformational changes, thus it cannot be definitively concluded at present that Trim28 is a direct molecular target of retinoic acid. Future studies will be conducted to further investigate this aspect.

This study has several limitations. Firstly, the mechanism by which Trim28 mediates the nuclear translocation of α-synuclein has not yet been fully elucidated; whilst our data confirm that Trim28 is an important factor, they do not clarify the specific transport mechanism. Secondly, the neuroprotective effects of retinoic acid were evaluated only in SH-SY5Y cells; no *in vivo* experiments were conducted to assess its neuroprotective effects following manganese exposure. Thirdly, whether Trim28 is a direct target of retinoic acid remains to be definitively confirmed, as CETSA cannot distinguish between direct binding and indirect conformational changes, and classical RAR/RXR-mediated signalling may account for some of the observed effects. Fourthly, our ChIP-qPCR analysis covered only two pairs of primers on the DAT promoter, and the available data merely indicate a correlation between H4K91ac and DAT transcription, rather than a causal relationship. Fifthly, there are significant differences between intraperitoneal manganese exposure and occupational inhalation exposure; consequently, the study results cannot be generalised to real-world occupational manganese exposure scenarios. Finally, the use of one-way ANOVA to test for pre-specified between-group differences did not formally establish interaction effects or capture longitudinal trends. Future research employing factorial or longitudinal designs will facilitate the resolution of these limitations.

**FIGURE 8:**
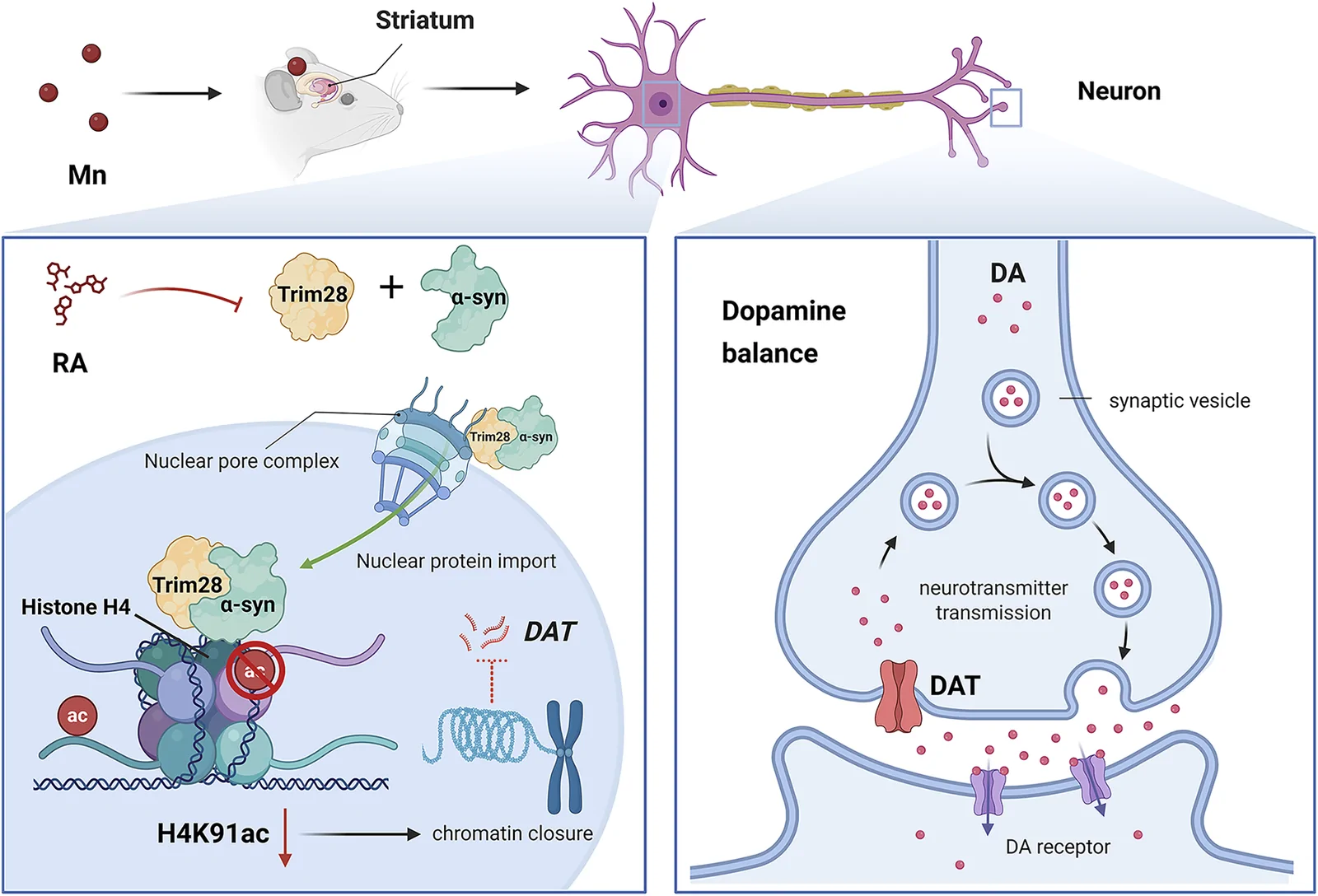
Schematic diagram illustrating the mechanism of action of the Trim28/α-syn/H4K91ac epigenetic axis in manganese-induced neurotoxicity in SH-SY5Y cells. Manganese mediates the translocation of α-syn to the nucleus via Trim28. Once in the nucleus, α-syn binds to histone H4, downregulates H4K91ac levels and inhibits DAT expression, thereby disrupting dopaminergic homeostasis. Retinoic acid, as an inhibitor of this pathway, reverses manganese-induced nerve cell damage by modulating Trim28. Created with BioRender.com.

In conclusion, manganese dysregulates histone acetylation and DA homeostasis via the Trim28/α-syn/H4K91ac axis, leading to neurotoxicity. Furthermore, this study suggests that Trim28 is a potential therapeutic target for retinoic acid (Figure 8).

## Data Availability

The original contributions presented in the study are included in the article/Supplementary Material, further inquiries can be directed to the corresponding authors.
