# Supplementary material for: Retinoic acid alleviates manganese-induced neurotoxicity in SH-SY5Y cells via the Trim28/α-synuclein/H4K91ac epigenetic axis
Source: Front Pharmacol. 2026 Aug 25;17:1883748. doi: 10.3389/fphar.2026.1883748 (PMC13550141; doi:10.3389/fphar.2026.1883748)
Supplement: Supplementary file 1 [file Supplementaryfile1.docx]

Supplementary Material

**Figure S1. Manganese reduces tyrosine hydroxylase (TH) expression in rats and cells.** (A) Immunohistochemical observation of TH expression in the striatum (x400), scale bar: 20 μm, black arrows indicate TH-positive expression. (B, C) Western blot analysis of TH protein levels in the striatum and quantitative analysis (n = 6). One-way ANOVA analysis of variance, followed by Tukey’s test, F (3,20) =13.79, *p*<0.0001. Control vs. Medium Mn:95% CI [0.02790, 0.3711]; Control vs. High Mn:95% CI [0.2257, 0.5689]. (D) RT-qPCR analysis of TH mRNA levels in cells (n = 3). One-way ANOVA analysis of variance, followed by Tukey’s test, F (3,8) =45.21, *p*<0.0001. 0 μmol/L Mn vs. 100 μmol/L Mn:95% CI [0.1275, 0.4959]; 0 μmol/L Mn vs. 200 μmol/L Mn:95% CI [0.3506, 0.7190]; 0 μmol/L Mn vs. 400 μmol/L Mn:95% CI [0.5193, 0.8877]. (E, F) Western blot analysis of TH protein levels in cells and quantitative analysis (n = 6). One-way ANOVA analysis of variance, followed by Tukey’s test, F (3,20) =47.74, *p*<0.0001. 0 μmol/L Mn vs. 100 μmol/L Mn:95% CI [0.1628, 0.4471]; 0 μmol/L Mn vs. 200 μmol/L Mn:95% CI [0.3267, 0.6110]; 0 μmol/L Mn vs. 400 μmol/L Mn:95% CI [0.5004, 0.7847]. Data are expressed as mean ± SEM. ∗*p*<0.05, ∗∗*p*<0.01, ∗∗∗*p*<0.001, ∗∗∗∗*p*<0.0001: statistically significant difference compared with the control group.

**Figure S2. Manganese reduces the levels of DAT transcription factors in cells.** (A, B) RT-qPCR detection of mRNA levels of DAT-specific transcription factors Nurr1 and Pitx3 in cells (n=3). One-way ANOVA analysis of variance, followed by Tukey’s test, F (3,8) =47.71, *p*<0.0001(nurr1); F (3,8) =16.63, *p*=0.0008(pitx3). 0 μmol/L Mn vs. 100 μmol/L Mn:95% CI [0.1717, 0.5335]; 0 μmol/L Mn vs. 200 μmol/L Mn:95% CI [0.3854, 0.7472]; 0 μmol/L Mn vs. 400 μmol/L Mn:95% CI [0.5235, 0.8853]（Nurr1）; 0 μmol/L Mn vs. 100 μmol/L Mn:95% CI [0.1006, 0.6700]; 0 μmol/L Mn vs. 200 μmol/L Mn:95% CI [0.1585, 0.7279]; 0 μmol/L Mn vs. 400 μmol/L Mn:95% CI [0.4041, 0.9735]（Pitx3） (C) Electrophoresis gel for RNA integrity analysis. (D, E) Hydrogen bonds formed between α-syn and H4, Trim28 in molecular docking. (F) Immunoprecipitation analysis of α-syn and Trim28 in the cell nucleus after manganese treatment. Data are expressed as mean ± SEM. ∗*p*<0.05, ∗∗*p*<0.01, ∗∗∗*p*<0.001, ∗∗∗∗*p*<0.0001: statistically significant difference compared with the control group.

**Figure S3. Screening of Si-RNA sequences and retinoic acid intervention time.** (A) Double immunofluorescence staining images of Trim28 and α-syn in cells. (B, C) Protein levels and quantification of α-syn after treatment with different Si-α-syn sequences (n=3). One-way ANOVA analysis of variance, followed by Tukey’s test, F (4,10) =34.25, *p*<0.0001. Control vs. Si-α-syn 2 :95% CI [0.2962, 0.6895]. Control vs. Si-α-syn 3 :95% CI [0.4550, 0.8483]. (D, E) Protein levels and quantification of Trim28 after treatment with different Si-Trim28 sequences (n=3). One-way ANOVA analysis of variance, followed by Tukey’s test, F (4,10) =23.01, *p*<0.0001. Control vs. Si-Trim28 1 :95% CI [0.1054, 0.3894]; Control vs. Si-Trim28 2 :95% CI [0.2912, 0.5752]; Control vs. Si-Trim28 3 :95% CI [0.02245, 0.3065]. (F) Effect of different retinoic acid concentrations on cell survival rate (n=3). One-way ANOVA analysis of variance, followed by Tukey’s test, F (5,12) =5.466, *p*=0.0075. 0 μmol/L Mn vs. 40 μmol/L RA:95% CI [0.7538, 28.38]. (G, H) Effect of retinoic acid at different time points on DAT protein levels in manganese-treated cells and quantification (n=3). One-way ANOVA analysis of variance, followed by Tukey’s test, F (5,12) =7.083, *p*=0.0027. Control vs. High Mn:95% CI [-0.5193, -0.03457]; High Mn vs. 72h(RA+Mn):95% CI [-0.5540, -0.06923]. Data are expressed as mean ± SEM. ∗*p*<0.05, ∗∗*p*< 0.01, ∗∗∗*p* < 0.001, ∗∗∗∗*p* < 0.0001: statistically significant difference compared with the control group. ^#^*P*<0.05, ^##^*p*<0.01, ^###^*p*<0.001, ^####^*p*<0.0001: Significant difference compared with the manganese exposure group.

**Fig. S4. Western blot used for statistical analysis in Figure 2 of the article.** (A, B, C, D) Western blot analyses of DAT and its internal control β-actin, H4K91ac and its internal control histone H4 in the striatum of manganese-exposed rats. (E, F, G, H) Western blot analyses of DAT and its internal control β-actin, H4K91ac and its internal control histone H4 in manganese-exposed cells.

**Fig. S5. Western blot used for statistical analysis in Figure 3 of the article.** (A, B) Western blot analysis of α-syn and the internal control β-actin in the striatum of manganese-exposed rats. (C, D) Western blot analysis of α-syn and the internal control β-actin in manganese-exposed cells. (E) Western blot analysis of α-syn and the internal controls β-tubulin and Lamin B1 in the cytoplasm and nucleus of cells exposed to manganese in vitro. (F)Co-immunoprecipitation analysis of α-syn and H4 in cell nuclei treated with manganese.

**Fig. S6. Western blot used for statistical analysis in Figure 4 of the article.**

**Fig. S7. Western blot used for statistical analysis in Figure 5 of the article.** (A) Western blot analysis of Trim28 and the internal control β-actin in the striatum of manganese-exposed rats. (B) Western blot analysis of Trim28 and the internal control β-actin in manganese-exposed cells. (C) Western blot analysis of Trim28 and the internal controls β-tubulin and Lamin B1 in the cytoplasm and nucleus of cells exposed to manganese in vitro. (D) Co-immunoprecipitation analysis of α-syn and Trim28 in cell cytoplasm treated with manganese. (E) Western blot results for Trim28, DAT, α-syn and the internal control β-actin, as well as H4K91ac and the internal control histone H4, following knockdown of Trim28.

**Fig. S8. Protein primitive bands used for statistical analysis in Figure 7 of the article.**

**Fig. S9. Western blot used for statistical analysis in Supplementary Figure S1.** (A) Western blot analysis of TH and the internal control β-actin in the striatum of manganese-exposed rats. (B) Western blot analysis of TH and the internal control β-actin in manganese-exposed cells.

**Fig. S10. Western blot used for statistical analysis in Supplementary Figure S2.**

**Fig. S11. Western blot used for statistical analysis in Supplementary Figure S3.** (A) Western blot analysis of α-syn following treatment with different siRNA sequences. (B) Western blot analysis of Trim28 following treatment with different siRNA sequences. (C) Western blot showing the effect of retinoic acid on DAT levels in manganese-treated cells at different time points.

**Fig.S12**. **Raw microscopic images from HE staining, transmission electron microscopy and immunohistochemistry.**

**Supplementary Table 1**

Table 1 Primer sequences used for quantitative real-time polymerase chain reaction (RT-qPCR)

| Gene name | Forward primer (5'->3') | Reverse primer (5'->3') | |
| --- | --- | --- | --- |
| *Human SNCA* | CATCGTTGGAACTACCAGAGTCACC | ACTCATTCCTCCTTCCTTCCTCACC | |
| *Human Trim28* | CTCGGAGCCTCTGTGTGAGA | TGCACACGCTTCTGTACGTC | |
| *Human SLC6A3* | AGGGAGCAGAGACGAAGACC | CAGCAGGTGCGTCTACAAGG | |
| *Human PITX3* | GAGCTAGAGGCGACCTTCCAGAG | TTGACCGAGTTGAAGGCGAATGG | |
| *Human NR4A2* | TGGCTGTTGGGATGGTCAAAGAAG | AGAATTGCTGGATATGCTGGGTGTC | |
| *Human GAPDH* | ATCCCTGAGCTGAACGGGAA | AGCGTCAAAGGTGGAGGAGT | |
| *Rat SNCA* | GGGCGTCCTCTATGTAGGTTCC | TGTCACTTGTTCTTTGGTCTTCTCAG | |
| *Rat Trim28* | CGAGATGGAGAACGAACAGTCTATTG | | TTGTGAGCATTGAGTTGGCAGTC |
| *Rat SLC6A3* | TTGGGTTTGGAGTGCTGATTGC | AGTTAATGGAGGTGGTGATGATTGC | |
| *Rat GAPDH* | ATGGGAAGCTGGTCATCAAC | GTGGTTCACACCCATCACAA | |

**Supplementary Table 2**

Table 2 Sequence of CHIP primers for DAT gene

| Gene name | Forward primer (5'->3') | Reverse primer (5'->3') |
| --- | --- | --- |
| *DAT(CHIP1)* | AATGGAAGAAATCCCGCCCG | TCGCACTCGCCTAAGAAAAC |
| *DAT(CHIP2)* | TTTCTTAGGCGAGTGCGAGG | CGAGGACTCTGCACGCTTC |

**Supplementary Table 3**

Table 3: Sequences of Trim28 and α-synuclein siRNA

| Gene name | Forward primer (5'->3') | Reverse primer (5'->3') | |
| --- | --- | --- | --- |
| Trim28-si-1 | CUGAGGACUACAACCUUAUTT | | AUAAGGUUGUAGUCCUCAGTT |
| Trim28-si-2 | GUGUGCAAGUGGAUGUCAATT | | UUGACAUCCACUUGCACACTT |
| Trim28-si-3 | GCAACGUACACAAGCAUGATT | | UCAUGCUUGUGUACGUUGCTT |
| SNCA-si-1 | CCAUCUCACUUUAAUAAUATT | | UAUUAUUAAAGUGAGAUGGTT |
| SNCA-si-2 | CUACGAACCUGAAGCCUAATT | | UUAGGCUUCAGGUUCGUAGTT |
| SNCA-si-3 | GCCACUGGCUUUGUCAAAATT | | UUUUGACAAAGCCAGUGGCTT |

Basis for manganese exposure doses in rats:

1.MnO_2_ occupational exposure limit（mg/m^3^）×respiratory volume for an occupational worker performing moderate physical labour for 8 hours (m^3^)÷Standard Adult Reference Weights(kg) ×Safety factor×Conversion factors for MnO₂ and MnCl₂·4H₂O =0.15×12÷60×100×2.276=6.828mg/kg

As 6.828 is the theoretical ‘no observed adverse effect level’ in rats, the lowest dose group was set at 6 mg/kg.

2. In this study, acute toxicity tests determined the median lethal dose (LD50) of MnCl₂·4H₂O in rats to be 147 mg/kg. As our study involved subchronic manganese exposure, we selected approximately one-eighth of the LD50, i.e. 18 mg/kg, as the high-dose group. Based on an arithmetic sequence, 12 mg/kg was selected as the medium-dose group.

3. Relevant literature was consulted.
